# Intracrine activity involving NAD-dependent circadian steroidogenic activity governs age-associated meibomian gland dysfunction

**DOI:** 10.1038/s43587-021-00167-8

**Published:** 2022-02-10

**Authors:** Lena Sasaki, Yuki Hamada, Daisuke Yarimizu, Tomo Suzuki, Hiroki Nakamura, Aya Shimada, Khanh Tien Nguyen Pham, Xinyan Shao, Koki Yamamura, Tsutomu Inatomi, Hironobu Morinaga, Emi K. Nishimura, Fujimi Kudo, Ichiro Manabe, Shogo Haraguchi, Yuki Sugiura, Makoto Suematsu, Shigeru Kinoshita, Mamiko Machida, Takeshi Nakajima, Hiroshi Kiyonari, Hitoshi Okamura, Yoshiaki Yamaguchi, Takahito Miyake, Masao Doi

**Affiliations:** 1 Department of Systems Biology, Graduate School of Pharmaceutical Sciences, Kyoto University, Kyoto, Japan; 2 Department of Ophthalmology, Kyoto Prefectural University of Medicine, Kyoto, Japan; 3 Department of Ophthalmology, Kyoto City Hospital, Kyoto, Japan; 4 Department of Ophthalmology, National Center for Geriatrics and Gerontology, Aichi, Japan; 5 Dpartment of Stem Cell Biology, Medical Research Institute, Tokyo Medical and Dental University, Tokyo, Japan; 6 Department of Disease Biology and Molecular Medicine, Graduate School of Medicine, Chiba University, Chiba, Japan; 7 Department of Biochemistry, Showa University School of Medicine, Tokyo, Japan; 8 Department of Biochemistry, Keio University School of Medicine, Tokyo, Japan; 9 Department of Frontier Medical Science and Technology for Ophthalmology, Kyoto Prefectural University of Medicine, Kyoto, Japan; 10 Senju Laboratory of Ocular Sciences, Senju Pharmaceutical Co., Kobe, Japan; 11 Laboratory for Animal Resources and Genetic Engineering, RIKEN Center for Biosystems Dynamics Research, Kobe, Japan; 12 Division of Physiology and Neurobiology, Graduate School of Medicine, Kyoto University, Kyoto, Japan

**Keywords:** Ageing, Endocrine system and metabolic diseases

## Abstract

Canonically, hormones are produced in the endocrine organs and delivered to target tissues. However, for steroids, the concept of tissue intracrinology, whereby hormones are produced in the tissues where they exert their effect without release into circulation, has been proposed, but its role in physiology/disease remains unclear. The meibomian glands in the eyelids produce oil to prevent tear evaporation, which reduces with aging. Here, we demonstrate that (re)activation of local intracrine activity through nicotinamide adenine dinucleotide (NAD^+^)-dependent circadian 3β-hydroxyl-steroid dehydrogenase (3β-HSD) activity ameliorates age-associated meibomian gland dysfunction and accompanying evaporative dry eye disease. Genetic ablation of 3β-HSD nullified local steroidogenesis and led to atrophy of the meibomian gland. Conversely, reactivation of 3β-HSD activity by boosting its coenzyme NAD^+^ availability improved glandular cell proliferation and alleviated the dry eye disease phenotype. Both women and men express 3β-HSD in the meibomian gland. Enhancing local steroidogenesis may help combat age-associated meibomian gland dysfunction.

## Abstract (web summary)

The authors show that eye-drop administration of an NAD^+^ precursor ameliorates age-associated meibomian gland dysfunction, a leading cause of dry eye in older individuals, by (re)activating intracrine steroidogenic activity.

## Main

Hormones are synthesized by endocrine organs, secreted into blood circulation and transported to distal target tissues, where they exert their effects. However, for steroids, in addition to the major steroidogenic endocrine organs, such as gonads and the adrenal gland, extragonadal non-endocrine tissues also participate in steroid hormone synthesis via an intracrine mechanism^1^. The hormones are produced locally in the same tissues where they act and are metabolically inactivated before release into the circulation^1,2^. The concept of intracrinology was established in the 1990s; however, its exact role in physiology and disease has not been fully explored.

Meibomian gland dysfunction (MGD) is the most common cause of dry eye, with incidence increasing with age^3,4^. Aging induces atrophic involution of the meibomian gland, a typical sign of MGD. However, no causal therapy exists owing to the lack of knowledge on the underlying molecular mechanism^5^. The enzyme 3β-HSD is essential for the biosynthesis of all classes of steroid hormones^6^ and has been shown to be expressed in the meibomian gland in humans;^7^ however, the molecular subtype of 3β-HSD protein expressed in this tissue is still undefined^8^. Here, we show that endogenous 3β-HSD activity declines with age, leading to MGD. We explore the physiological role of 3β-HSD-mediated local intracrine activity and demonstrate its potential for treating MGD and MGD-associated dry eye disease.

## Results

### Age-associated meibomian gland atrophy

**Fig. 1:**
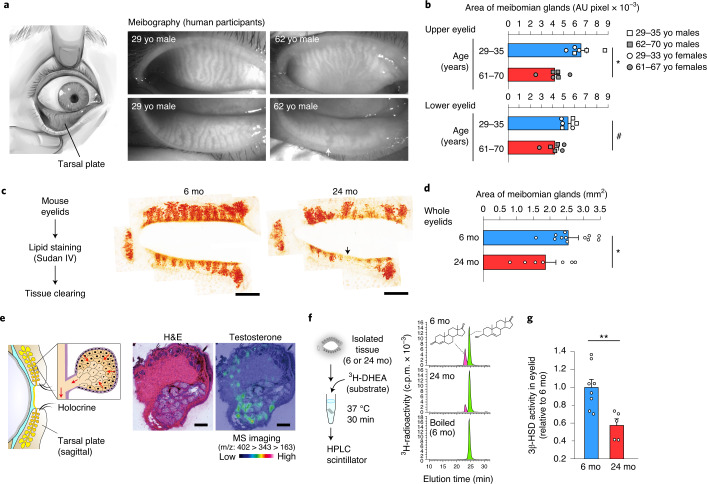
Age-associated meibomian gland atrophic changes in humans and mice. **a**, Schematic of human eye emphasizing the tarsal plate. Infrared meibography images of upper and lower eyelids of young (29 years) and aged (62 years) men. yo, years old. Arrow indicates area of meibomian gland loss (right). **b**, Quantification of meibomian gland areas in upper and lower eyelids from nonpathological young (29–35 years; three men, three women) and aged (61–70 years; three men and three women) participants. ^*^P = 0.0043 (top); ^#^*P* = 0.0165 (bottom). AU, arbitrary unit. **c**, Outline of whole-mount meibomian gland staining of mouse eyelids. Pictures are representative of mouse upper and lower eyelids at 6 and 24 months of age. mo, months old. Arrow: area of meibomian gland loss. Scale bars, 1 mm. **d**, Quantification of meibomian gland areas of young (6 months, *n* = 12) and aged (24 months, *n* = 7) mice. ^*^P = 0.0213. **e**, Local testosterone distribution in the meibomian gland using MS. Mouse eyelids were cut in a sagittal plane and counterstained with hematoxylin and eosin (H&E). Scale bar, 200 µm. Data are representative of four biologically independent eyelid samples with similar results. **f**, Outline of 3β-HSD activity measurements in the meibomian gland. Chromatograms show endogenous 3β-HSD activity arising from the meibomian gland of young (6 months) and aged (24 months) mice. 3β-HSD enzymatic activities were determined by measuring the conversion of ^3^H-DHEA (green) to ^3^H-androstenedione (magenta). c.p.m., counts per minute. **g**, Relative 3β-HSD activity in (**f**). Plots represent biologically independent samples of young (6 months, *n* = 8) and aged (24 months, *n* = 5) mice. ^**^P = 0.0056. Data are the mean ± s.e.m. and were analyzed using unpaired two-sided Student’s *t*-test (**b**,**d**,**g**).

We performed noncontact infrared meibography of the upper and lower eyelids of young (29–35 years) and aged (61–70 years) men and women (Fig. 1a and Supplementary Fig. 1) and found a significant age-associated reduction in gland size in both upper and lower eyelids, with age-associated morphological changes, characterized by tubular shortening and thinning and gland dropout (Fig. 1a, arrow), which are atrophic features widely observed in aged humans^3,4^ (Fig. 1a,b and Supplementary Fig. 1). Consistently, aged mice displayed meibomian gland atrophy (Fig. 1c,d; 24 months old versus 6 months old). Morphologically, gland shortening, thinning and dropout were also observed in aged mice (Fig. 1c).

**Extended Data Fig. 1:**
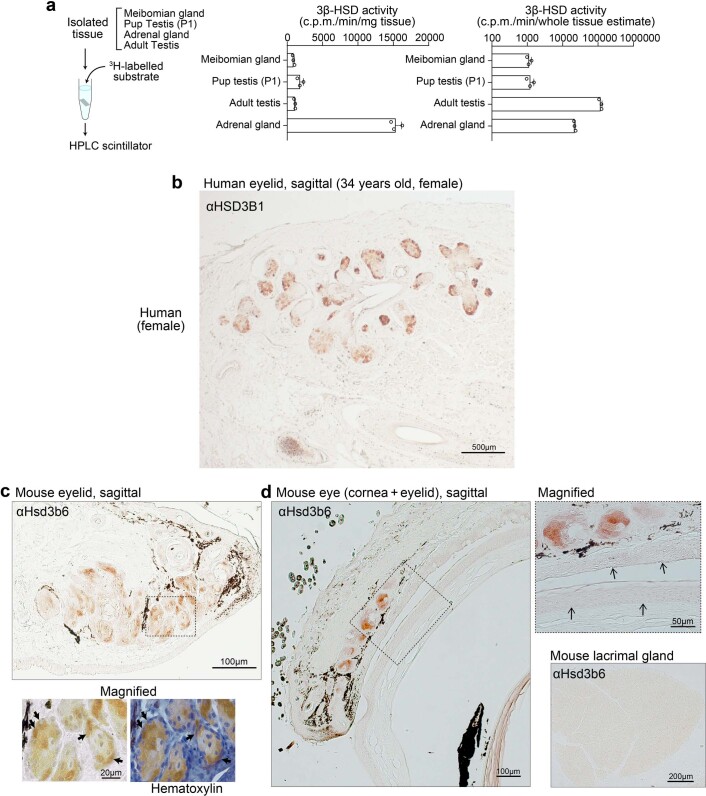
Meibomian gland 3β-HSD activity and protein localization. **a**, Relative intensity of local 3β-HSD activity of pup testis, adult meibomian gland, testis, and adrenal gland, related to Fig. 1f. Tissues were isolated from 3-month-old male mice except pup testis, which was isolated from P1 newborn mice. 3β-HSD activities were measured as described in Fig. 1f. Data are the mean ± SEM (*n* = 3 biologically independent animals for all groups). **b**, **c**, Immunolocalization of the meibomian-specific 3β-HSD enzyme in human and mouse, related to Fig. 2a-c.**b**, A human female eyelid section, immunolabeled for HSD3B1. Bar, 500 µm. Similar results were obtained from three independent experiments. **c**, Immunolocalization of Hsd3b6 in the mouse eyelid. A dotted box indicates the area of high magnification view. Arrows, Hsd3b6-immunopositive acinar cells in the meibomian gland, before and after hematoxylin counterstaining. **d**, Anti-Hsd3b6 immunostaining of mouse corneal plus eyelid section and extraorbital lacrimal gland section. Note that little or no appreciable Hsd3b6 immunopositive staining was observed in the cornea, lacrimal gland, or conjunctiva. Scale bars are shown on the images. Immunohistology data in **b**–**d** were reproducibly observed in three independent experiments.

Intracrine activity has been proposed in the meibomian glands of both humans and mice, owing to the abundant expression of enzymes required for steroidogenesis^4^. In support of this intracrine model, we revealed, using matrix-assisted laser desorption/ionization (MALDI)-imaging mass spectrometry (MS), that testosterone is enriched in the meibomian gland (Fig. 1e and Supplementary Fig. 2). Freshly isolated mouse meibomian glands, incubated in vitro in a buffer containing 3β-HSD substrate (1,2,6,7-^3^H-DHEA; hereafter, ^3^H-DHEA), displayed marked 3β-HSD activity (Fig. 1f), which was nullified by boiling the tissue. This activity in the meibomian gland was almost comparable to that in a pup testis (Extended Data Fig. 1a). We found that meibomian gland 3β-HSD activity was significantly reduced (by ~50%) in aged mice compared to that in young mice (Fig. 1g).

### Ablation of meibomian steroidogenesis

**Fig. 2:**
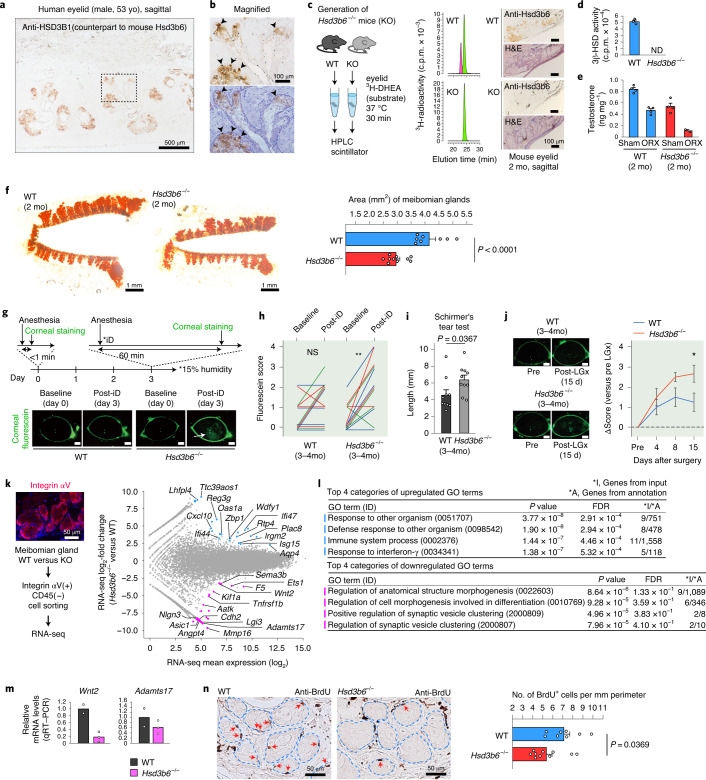
Meibomian gland atrophy and associated EDE in *Hsd3b6*^−/−^ mice. **a**, Human eyelid, immunolabelled for HSD3B1. **b**, Magnified view of **a**. Arrowheads, HSD3B1-positive acinar cells. **c**, Chromatograms showing loss of 3β-HSD activity in *Hsd3b6*^−/−^ mice. Anti-Hsd3b6 immunohistochemistry−H&E staining of serial sections (right). KO, knockout. **d**, 3β-HSD activities in **c**. *n* = 3 mice for both genotypes. ND, not detectable. **e**, Meibomian gland tissue testosterone concentrations with or without orchidectomy (ORX). *n* = 4 mice per group except *n* = 2 for *Hsd3b6*^−/−^ ORX. **f**, Whole-mount meibomian gland staining and quantification of gland area in WT (*n* = 8) and *Hsd3b6*^−/−^ (*n* = 10) mice. **g**, Schematic of EDE test. Arrow indicates area of the corneal epithelial defect. Scale bars, 0.5 mm. **h**, Pair-wise comparison of epithelial defect score in **g**. WT, *n* = 13; *Hsd3b6*^−/−^, *n* = 13 mice. ^**^P = 0.0025, three-way ANOVA with Sidak test. NS, not significant. **i**, Measurements of tear quantity. *n* = 10 mice per genotype. **j**, Change in fluorescein infiltration score at 4, 8 and 15 d after lacrimal gland excision, relative to previous surgery. WT, *n* = 8; *Hsd3b6*^−/−^, *n* = 6 mice. Bars, 0.5 mm. ^*^P = 0.0340. **k**, FACS/RNA-seq data in MA-plot format (*n* = 2 biologically independent cell pools for both genotypes); significantly upregulated and downregulated transcripts (DESeq2 *P*_adj_ < 0.1) are colored blue and pink, respectively. **l**, The top four GO terms of the genes up- or downregulated with *Hsd3b6* ablation. *P* values were calculated by GOrilla. FDR, false discovery rate. **m**, Quantitative PCR with reverse transcription (qRT–PCR) validation of reduced *Wnt2* and *Adamts17* mRNA levels in the *Hsd3b6*^−/−^ meibomian gland. WT, *n* = 2; *Hsd3b6*^−/−^, *n* = 3 biologically independent cell pools. **n**, Reduced BrdU incorporation in *Hsd3b6*^−/−^ mice. Dashed lines indicate meibomian acini. Arrows indicate BrdU^+^ cells. The graph shows the numbers of BrdU-labeled cells per mm perimeter of the acini. WT, *n* = 8; *Hsd3b6*^−/−^, *n* = 10 mice. Error bars indicate s.e.m. Immunohistology data in **a**–**c**,**k** were reproducibly obtained in four independent experiments. Statistics in **f**,**i**,**j** were two-way analysis of variance (ANOVA) and Bonferroni test; statistical test in **n** was unpaired, two-sided Student’s *t*-test.

Using human eyelid specimens, we demonstrated that the human 3β-HSD isozyme protein that is expressed in the meibomian gland cells is type I, namely HSD3B1, a subtype different from that mainly expressed in the gonads and adrenal gland (HSD3B2), in both male and female (Fig. 2a,b and Extended Data Fig. 1b). Similar subtype specificity was also observed for mice, in which Hsd3b6 (a counterpart of human HSD3B1) is expressed in the meibomian gland^9^, whereas Hsd3b1 (a counterpart of human HSD3B2) is expressed in the gonads and adrenal gland^6,10^ (Extended Data Fig. 1c and Fig. 2c). Immunohistochemically, Hsd3b6 expression was restricted to the meibomian gland, with little or no appreciable expression in the cornea, lacrimal gland or conjunctiva (Extended Data Fig. 1c,d).

**Extended Data Fig. 2:**
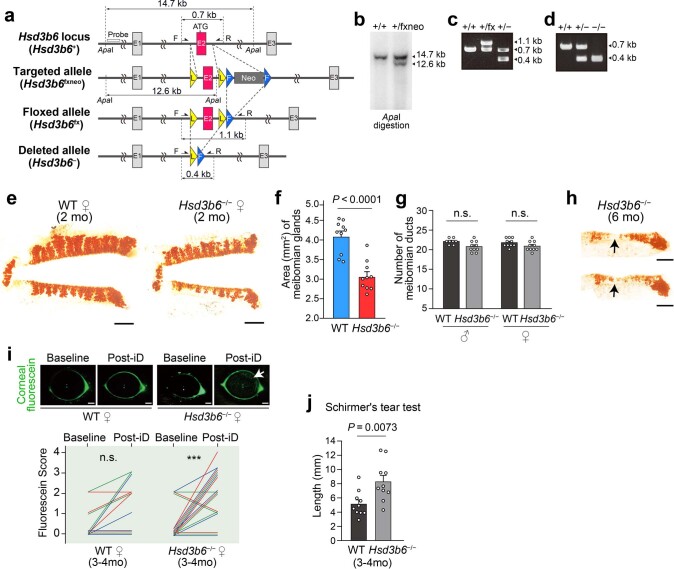
Generation of *Hsd3b6*^−/−^ mice and phenotypic analysis of the meibomian gland function with the female mice, related to Fig. 2c-i. **a**, Schematic structure of the wild-type (*Hsd3b6*^+^), targeted (*Hsd3b6*^fxneo^), floxed (*Hsd3b6*^fx^) and deleted (*Hsd3b6*^−^) allele of the mouse type IV 3β-HSD encoding gene. The second exon of the gene, which contains the initiation codon (ATG), was flanked with loxP sites. **b**, Southern blot of *Apa*I-digested DNA from *Hsd3b6*^+/+^ and *Hsd3b6*^+/fxneo^ mice in (**a**). **c**, **d**, Genotyping PCRs. Amplified DNA fragments from *Hsd3b6*^+^ (0.7 kb), *Hsd3b6*^fx^ (1.1 kb), and *Hsd3b6*^−^ (0.4 kb) alleles are indicated. **e**, Whole-mount meibomian gland staining of WT and *Hsd3b6*^−/−^ female mice. Bars, 1 mm. **f**, Quantification of the meibomian gland area in (**e**) (2 mo, *n* = 10 mice per genotype). Male data are shown in Fig. 2f. **g**, The number of meibomian gland ducts. *n* = 8, WT male; *n* = 10, *Hsd3b6*^−/−^ male; *n* = 10, WT female; *n* = 10, *Hsd3b6*^−/−^ female mice. **h**, Representative area of gland drop-out of 6-mo *Hsd3b6*^−/−^ mouse lower eyelids (*n* = 2 mice). **i**, Evaporative dry-eye test for female mice. Photos show representative images of corneal fluorescein staining before and after desiccation (iD). Bars, 0.5 mm. Arrow indicates the area of the epithelial defect. The graphs represent pair-wise comparison of corneal fluorescein staining scores before and after iD of WT and *Hsd3b6*^−/−^ female mice (3-4 mo, *n* = 12, WT; *n* = 18, *Hsd3b6*^−/−^). Male data are shown in Fig. 2h. ^***^P = 0.0006. **j**, Schimer’s tear test of WT and *Hsd3b6*^−/−^ female mice (*n* = 10, for each genotype). Male data are available in Fig. 2i. Error bars indicate SEM. Statistics in **f**-**g** and **j**: two-way ANOVA, Bonferroni test. Gel images shown in **b**-**d** are representative of three independent experiments with similar results. Data in **h** were reproducibly observed in four independent animals. n.s., not significant. mo, months old.

We developed *Hsd3b6*^−/−^ mice (Extended Data Fig. 2), the first 3β-HSD-knockout animals generated, and confirmed the complete abolition of anti-Hsd3b6 immunoreactivity in the meibomian gland (Fig. 2c). *Hsd3b6*^−/−^ mice are viable and fertile. Freshly isolated whole meibomian glands did not display detectable 3β-HSD activity, demonstrating that Hsd3b6 is the sole enzyme responsible for the entire 3β-HSD activity in the meibomian gland (Fig. 2c,d). This local 3β-HSD activity was essential for maintaining normal testosterone levels in the meibomian gland (Fig. 2e); as revealed by liquid chromatography (LC)–MS/MS, tissue testosterone levels decreased to ~50% with Hsd3b6 loss and to ~10% post-castration, revealing that both locally produced testosterone and circulating testosterone supplied by the testes (in which the other 3β-HSD subtype operates) almost equally contribute to its levels in the meibomian gland.

### Intracrine deficiency causes MGD

**Extended Data Fig. 3:**
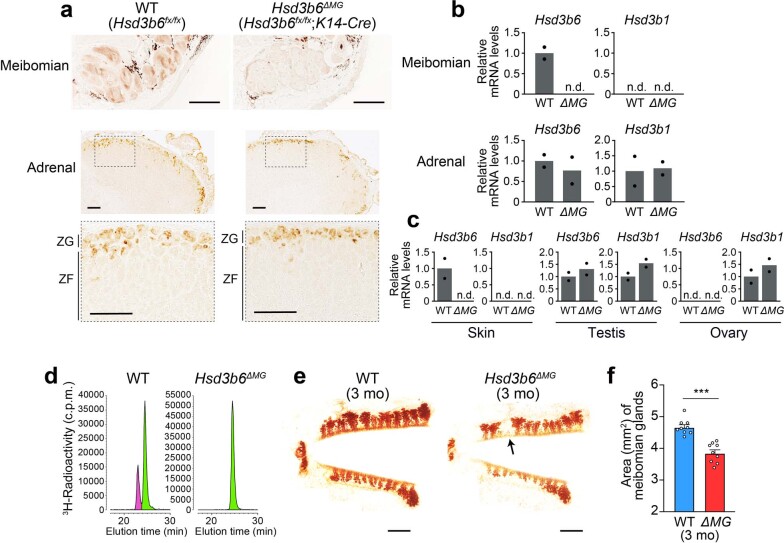
The loss of tissue-specific Hsd3b6 activity of conditional *Hsd3b6*^ΔMG^ mice causes atrophy of the meibomian gland. **a**, Immunohistological validation of Hsd3b6 protein expression in the meibomian gland and adrenal gland of WT (*Hsd3b6*^fx/fx^) and *Hsd3b6*^ΔMG^ (*Hsd3b6*^fx/fx^;K14-Cre) mice. ZG, zona glomerulosa. ZF, zona fasciculata. Scale bars, 100 µm. Data are representative of three independent mice with similar results. **b**, The mRNA levels of *Hsd3b6* and *Hsd3b1* in the meibomian gland and adrenal gland of WT and *Hsd3b6*^ΔMG^ mice (*n* = 2 biologically independent samples, for each tissue). n.d., not detectable. **c**, The mRNA levels of *Hsd3b6* and *Hsd3b1* in the skin, testis, and ovary of WT and *Hsd3b6*^ΔMG^ mice (*n* = 2 biologically independent samples, for each tissue). Male mice were used for analysis except for the ovary. Values were determined by qRT-PCR and normalized to those of ribosomal protein large P0 gene. n.d., not detectable. **d**, Chromatograms showing the loss of local 3β-HSD activity in the meibomian gland of *Hsd3b6*^ΔMG^ mice. 3β-HSD enzymatic activities in WT and *Hsd3b6*^ΔMG^ eyelids were determined by measuring the conversion of ^3^H-DHEA (green) to ^3^H-androstendione (magenta). **e**, Representative images of whole-mount meibomian gland staining of upper and lower eyelids of WT and *Hsd3b6*^ΔMG^ mice. mo, months old. Arrow indicates the area of gland dropout. Scale bars, 1 mm. **f**, Quantification of the area of the meibomian glands of WT and *Hsd3b6*^ΔMG^ mice (*n* = 9 mice for each genotype, 3 mo). ^***^P < 0.0001, unpaired two-sided *t*-test. All values are presented as mean ± SEM.

Whole-mount morphologies of the meibomian glands of *Hsd3b6*^−/−^ mice revealed severe atrophy of the meibomian gland by 2 months of age, compared to that in age-matched wild-type (WT) mice (Fig. 2f), with no difference in the number of meibomian ducts between genotypes (Extended Data Fig. 2g). Exacerbated meibomian atrophy, including gland dropout, was observed by 6 months of age in *Hsd3b6*^−/−^ mice (Extended Data Fig. 2h), but not in age-matched WT mice (Fig. 1c). There were no sex-related differences in the data (Extended Data Fig. 2e). To specify the role of Hsd3b6 in the meibomian gland, we developed *Hsd3b6*^ΔMG^ mice by crossing floxed *Hsd3b6* mice with mice expressing Cre recombinase under the keratin 14 (K14) promoter^11^. Immunohistochemistry revealed the abolition of Hsd3b6 in the meibomian gland, but not in the adrenal zona glomerulosa cells, where this enzyme is involved in aldosterone production^10^ (Extended Data Fig. 3a). Quantitative real-time PCR analysis further validated the ablation of *Hsd3b6* messenger RNA expression in the meibomian gland and skin, but not in the adrenal gland and testis (Extended Data Fig. 3b,c). Expression of the paralogous subtype *Hsd3b1* remained unaltered in the testis, adrenal and ovary, consistent with the hypothesis that steroidogenic activities in these organs are not affected by *Hsd3b6* ablation. The meibomian glands of *Hsd3b6*^ΔMG^ mice had no detectable 3β-HSD activity and showed a significant reduction in size (Extended Data Fig. 3d−f). These data support a causative role of 3β-HSD activity in maintaining normal meibomian gland morphology.

In MGD, decreased meibomian oil production enhances tear fluid evaporation at the ocular surface, leading to evaporative dry eye (EDE) disease^3,4^. Using our mouse model, we established this pathological connection (Fig. 2g−j). To score EDE incidence, mice were anesthetized and eyes were exposed to air for 1 h. We scored ocular surface impairment by corneal fluorescein staining before and after this imposed desiccation (iD). Minimal punctate staining was observed before iD, regardless of genotype (Fig. 2g). However, in *Hsd3b6*^−/−^ mice, fluorescein scores significantly increased after 1 h of iD compared to that before treatment, for both males and females (males, Fig. 2h; females, Extended Data Fig. 2i). In WT mice, although fluorescein scores tended to increase following iD, these differences were not significant for males or females (Fig. 2h and Extended Data Fig. 2i). *Hsd3b6*^−/−^ mice, despite exhibiting the EDE phenotype, had a greater tear volume than WT mice (for males, Fig. 2i; for females, Extended Data Fig. 2j), in Schirmer’s test, suggesting a compensatory tear production to alleviate the dry eye phenotype^12^. Congruently, a surgical removal of the lacrimal gland led to a more obvious EDE phenotype in *Hsd3b6*^−/−^ mice than WT mice (Fig. 2j).

Next, we performed RNA-sequencing (RNA-seq) analysis using fluorescence-activated cell sorting (FACS)-purified meibomian gland cells. Integrin αV (Itgav) was identified as a useful cell-surface marker for the acinar cells (Fig. 2k and Supplementary Fig. 3). MA-plot representations of *Hsd3b6*^−/−^ versus WT expression data indicate significantly upregulated and downregulated transcripts (DESeq2, *P*_adj_ < 0.1) in blue and pink, respectively (Fig. 2k). As revealed by Gene Ontology (GO) analysis (Fig. 2l and Supplementary Data 1), cells sorted from *Hsd3b6*^−/−^ mice showed increased expression of inflammation-related genes, including *Ifi47*, *Irgm2*, *Lhfpl4*, *Oas1a*, *Plac8*, *Reg3g*, *Rtp4*, *Ttc39aos1*, *Wdfy1* and *Zbp1*, a possible pathological consequence of deficient local steroidogenesis and MGD^13,14^. On the other hand, genes suppressed in *Hsd3b6*^−/−^ mice exhibited the highest enrichment score for the GO terms ‘regulation of anatomical structure morphogenesis’ and ‘regulation of cell morphogenesis involved in differentiation’ (Fig. 2l), indicating a more direct signature to explain the meibomian gland atrophy. We observed reduced expression of *Wnt2* and *Adamts17*, which encode a morphogen of the Wnt/β-catenin pathway^15^ and a member of the ADAMTS family of secreted metalloproteases involved in tissue development and remodeling^16^, respectively, among other morphogenic genes, such as *Aatk*, *Angpt4*, *Cdh2*, *Ets1*, *Kif1a*, *Mmp16*, *Nlgn3*, *Sema3b* and *Tnfrsf1b* (Fig. 2k,m). Consistently, meibomian glands of *Hsd3b6*^−/−^ mice exhibited significantly reduced cell proliferation, compared to WT mice (Fig. 2n). Actively dividing cells, which were labeled by 5-bromodeoxyuridine (BrdU), were mostly located in the basal acinar cell layer of the meibomian gland (Fig. 2n), consistent with continuous cell supply required for the holocrine activity of this tissue (Fig. 1e and Supplementary Fig. 4)^3,17^.

### Circadian clock and 3β-HSD activity

**Fig. 3:**
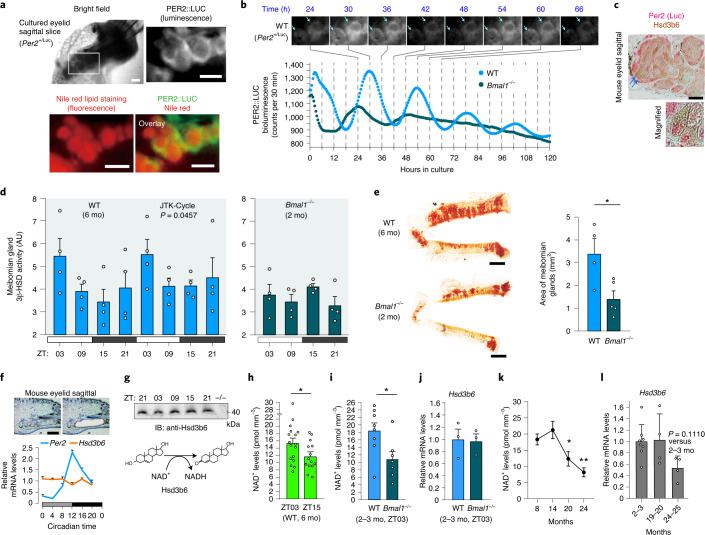
Circadian 3β-HSD enzymatic activity and age-related decline of its coenzyme NAD^+^ in the meibomian gland. **a**, Organotypic eyelid slice from *Per2*^Luc/+^ mice and magnified images of PER2::LUC luminescence with Nile red staining. Scale bars, 100 μm. **b**, Circadian oscillation of PER2::LUC. Arrows indicate representative rhythmic, basal cell layers. *Bmal1*^−/−^;*Per2*^Luc/+^ eyelid slice failed to keep oscillation. **c**, Dual immunolabelling of PER2::LUC and Hsd3b6. Arrows indicate representative double-labeled cells. Scale bar, 50 μm. **d**, Time-of-day-dependent changes in 3β-HSD activity in WT and *Bmal1*^−/−^ meibomian gland (*n* = 4 mice per time point). **e**, Quantification of meibomian gland area in WT (*n* = 4) and *Bmal1*^−/−^ (*n* = 5) mice. ^*^P = 0.0229. **f**, 24 h mRNA expression of *Per2* and *Hsd3b6* in laser-microdissected meibomian gland (*n* = 4 mice per data point). Scale bar, 200 µm. **g**, 24 h temporal variations in Hsd3b6 protein expression. Immunoreactivities were abolished in *Hsd3b6*^−/−^ mice. Requirement of NAD^+^ for dehydrogenase activity of Hsd3b6 (bottom). IB, immunoblot. **h**, NAD^+^ levels in the meibomian gland at ZT03 and ZT15. *n* = 17, ZT03; *n* = 14, ZT15; biologically independent laser-microdissected tissue samples. ^*^P = 0.0477. **i**, Meibomian gland NAD^+^ levels in WT (*n* = 8) and *Bmal1*^−/−^ (*n* = 7) mice. ^*^P = 0.0261. **j**, Relative mRNA levels of *Hsd3b6* in laser-microdissected meibomian gland, normalized to those of *Itgav* in WT and *Bmal1*^−/−^ mice. *n* = 3 mice per group. **k**, Meibomian NAD^+^ levels at 8, 14, 20 and 24 mo. *n* = 8 WT mice per group except *n* = 7 for 24 mo. ^*^P = 0.0301; ^**^P = 0.0011, versus 14 mo. **l**, *Hsd3b6* mRNA levels at 2–3, 19–20 and 24–25 months of age. *n* = 8, 2–3 mo; *n* = 4, 19–20 mo; *n* = 4, 24–25 mo mouse meibomian glands. Values are presented as mean ± s.e.m. Statistics in **k**,**l** were one-way ANOVA and Bonferroni test; statistical test in **e**,**h**,**i** was unpaired, two-sided Student’s *t*-test. Data presented in **a**–**c**,**g** are representative of four biologically independent experiments with similar results.

We next investigated the manner in which meibomian gland intracrine activity is regulated. We found that basal acinar cells of the meibomian gland have an autonomous circadian clock (Fig. 3a−c), displaying circadian 3β-HSD activity (Fig. 3d). Time-lapse bioluminescence microscopy of isolated eyelids from *Per2*^Luc/+^ reporter mice^18^, which facilitate the analysis of circadian expression dynamics of the key clock component Per2 in tissues and cells^19^, revealed abundant and robustly cycling Per2-Luc luminescence in the rims of meibomian gland acini (Fig. 3a,b). Immunohistochemistry further confirmed colocalization of Per2 and Hsd3b6 in basal acinar cells (Fig. 3c), whereas Per2 immunostaining signals were predominantly nuclear (Supplementary Fig. 5a)^20^ and Hsd3b6 signals were perinuclear (Extended Data Fig. 1c)^21^. Western blot also confirmed high-amplitude Per2 oscillation in the gland (Supplementary Fig. 5b). To test for a rhythm in 3β-HSD activity, meibomian glands were sampled from mice at distinct time points over two consecutive days. Meibomian gland 3β-HSD activities cycled on a daily basis, with peaks at the early phase of the light period, zeitgeber time (ZT)03 (ZT00 and ZT12 refer to light on and off times, respectively; Fig. 3d). In circadian clock-deficient *Bmal1*^−/−^ mice^22^, 3β-HSD activity was constantly low, with no significant elevation at ZT03 (Fig. 3d), indicating that the core clock machinery is required for the daily 3β-HSD activation. Marked meibomian gland tissue atrophy was also observed in *Bmal1*^−/−^ mice (Fig. 3e), possibly due to the constantly attenuated arrhythmic activity of 3β-HSD.

### Meibomian NAD^+^ availability

**Extended Data Fig. 4:**
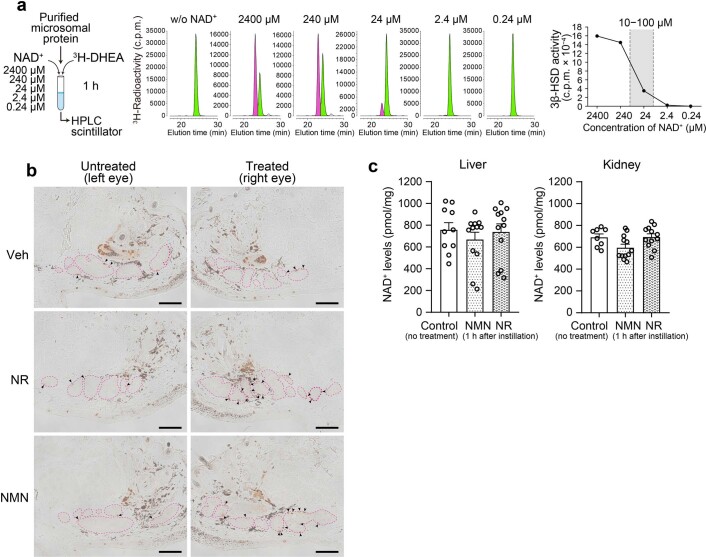
Effects of NAD^+^ augmentation in vitro and in vivo, related Fig. 4a,f,k. **a**, 3β-HSD cofactor kinetics. Purified microsomal protein fraction from the meibomian gland was incubated in vitro with different doses of NAD^+^. Chromatograms show NAD^+^ concentration-dependent 3β-HSD enzymatic activity. 3β-HSD activities were assessed as described in Fig. 1f. A drastic change in 3β-HSD activity was observed in a physiological range of intracellular NAD^+^ variation in mammalian cells (~10–100 µM). **b**, Representative immunohistochemistry images for BrdU incorporation assays shown in Fig. 4f. Dashed lines: meibomian gland acini. Arrowheads; representative BrdU-immunolabeled cells. Scale bars, 100 µm. Data are representative of mice independently treated with Veh (*n* = 7), NR (*n* = 6), or NMN (*n* = 6). mo, months old. **c**, NAD^+^ levels in the liver and kidney 1 h after single eye drop administration of NMN and NR, related to Fig. 4k. 18-month-old mice were used. Mice received 5 μL of 5% NMN or NR-containing phosphate buffered saline to the right eye. Data were normalized to tissue weight. Error bars show SEM; *n* = 12 mice per group, except *n* = 10 for liver control and *n* = 8 kidney control.

Despite the daytime-dependent variations and age-related decline in 3β-HSD activity, *Hsd3b6* expression did not diurnally fluctuate, was not reduced by *Bmal1* deficiency and did not decrease with aging (Fig. 3f,j,l). Western blot analysis also revealed a lack of daily variation in Hsd3b6 levels (Fig. 3g). Therefore, we considered the possibility that catalytic 3β-HSD activity is defined not only by the amount of enzyme, but also by the abundance of its cofactor NAD^+^ (Fig. 3g). The 3β-HSD Michaelis constant (*K*_M_) for NAD^+^ (34 μM for HSD3B1 and 86 μM for HSD3B2)^23^ falls within its known cellular concentration range^24,25^. The mouse meibomian gland 3β-HSD *K*_M_ was also approximately 20 μM (Extended Data Fig. 4a). In addition, LC–MS/MS analysis measuring meibomian gland NAD^+^ revealed NAD^+^ levels of 15.4 ± 1.3 and 11.7 ± 1.0 pmol mm^−3^ of tissue volume at ZT03 and ZT15, the times of maximal and minimal 3β-HSD activity, respectively (mean ± s.e.m.; Fig. 3h). *Bmal1* deficiency caused a significant reduction in meibomian NAD^+^ from 18.4 ± 2.1 to 10.8 ± 2.0 pmol mm^−3^ (*P* = 0.0261; Fig. 3i). Meibomian NAD^+^ levels started to decline after 14 months and were at 12.3 ± 2.2 and 8.2 ± 1.3 pmol mm^−3^ at 20 and 24 months, respectively (*P* = 0.0301, 20 versus 8 months, *P* = 0.0011, 24 versus 8 months; Fig. 3k). These data support the hypothesis of altered 3β-HSD activity due to limited coenzyme NAD^+^ availability in the meibomian gland.

### NAD^+^ repletion ameliorates MGD

**Fig. 4:**
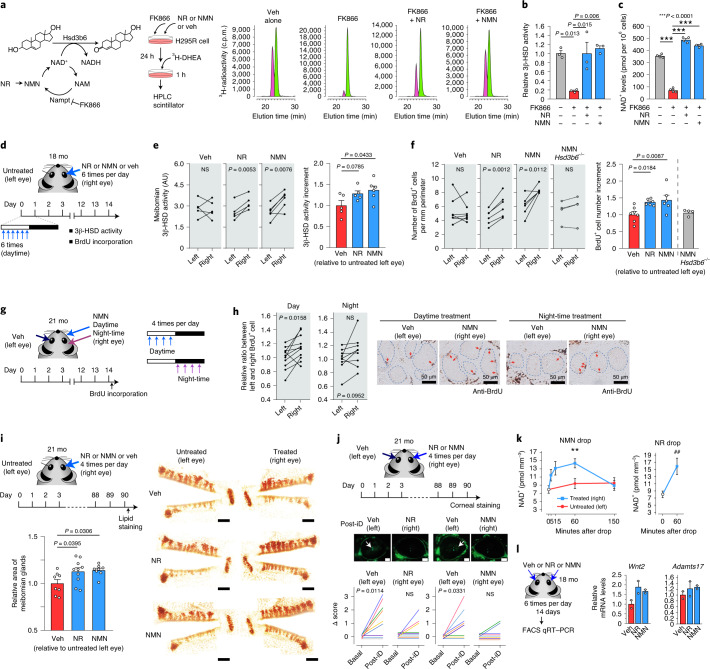
Eye-drops of NAD^+^ precursors restore 3β-HSD activity and improve meibomian gland function in aged mice. **a**, Chromatograms showing NAD^+^-dependent 3β-HSD enzymatic activity in H295R cells. Veh, vehicle. **b**, 3β-HSD activities in **a**. *n* = 3 biologically independent cell samples for each treatment. **c**, Intracellular NAD^+^ levels in **a**. *n* = 4 cell samples, except *n* = 3 for NMN. **d**, Schematic of eye-drop regimen. Left eye was untreated. **e**, Meibomian 3β-HSD activities in **d**. Veh, *n* = 5; NR, *n* = 5; NMN, *n* = 6 mice. **f**, BrdU^+^ acinar cells in **d**. Veh, *n* = 7; NR, *n* = 6; NMN, *n* = 6 mice. For comparison, *Hsd3b6*^−/−^ mice were treated with NMN (*n* = 4, 2–3 mo). **g**, Schematic of daytime versus night-time regimen. **h**, Relative ratio between left and right eye BrdU^+^ cells in **g**. *n* = 11 (daytime) or 9 (night-time) mice. Arrows indicate BrdU^+^ acinar cells. **i**, Meibomian gland morphology after 90-d instillation of Veh, NR or NMN. Scale bars, 1 mm. The graph shows fold increases in gland area relative to that in untreated left eyes. Veh, NMN, *n* = 8; NR, *n* = 10 mice. **j**, EDE test after 90-d instillation. NR, *n* = 9; NMN, *n* = 8 mice. Left eye was treated with Veh. Arrows indicate area of the epithelial defect. Scale bars, 0.5 mm. The graphs represent Δscore before and after desiccation (iD). **k**, Meibomian NAD^+^ levels after single eye-drop (*n* = 8 eyes per time point, except *n* = 7 for 60 min, NMN, right, *n* = 6 for 150 min, NMN (right) and *n* = 6 for NMN (left)). ^**^P = 0.0072, two-way ANOVA and Bonferroni test; ^##^*P* = 0.0068, unpaired two-sided Student’s *t*-test. **l**, Relative mRNA levels of *Wnt2* and *Adamts17* after 14-d instillation of Veh, NR or NMN. Values are mean ± variance of two biological replicates. Error bars in **b**,**c**,**e**,**f**,**i**,**k** indicate s.e.m. Statistics in **b**,**c**,**i** were one-way ANOVA and Bonferroni test; statistics in **e**,**f** (right) were one-way ANOVA and Holm–Sidak test; statistics in **e**,**f** (left), **h**,**j** were paired, two-sided Student’s *t*-tests.

Arguing further that NAD^+^ bioavailability is a determinant of intracellular 3β-HSD activity, FK866, an inhibitor of the rate-limiting enzyme of NAD^+^ synthesis^24^ attenuated endogenous 3β-HSD activity in cultured human steroidogenic cells (H295R) (Fig. 4a−c). Exogenous supplementation with an NAD^+^ bioprecursor, either nicotinamide mononucleotide (NMN) or nicotinamide riboside (NR), which bypass the rate-limiting step of the salvage NAD^+^ pathway^24^, restored both NAD^+^ levels and 3β-HSD activity (Fig. 4b,c), suggesting that NAD^+^ repletion can improve 3β-HSD activity.

Restoration of local intracrine activity might ameliorate MGD. We sought to boost local intracrine activity via topical eye-drop administration of NAD^+^ precursors (Fig. 4d). NMN or NR-containing eye drop or placebo (vehicle) was administered to the right eyes of aged mice six times per day during daytime, when endogenous 3β-HSD is activated (Fig. 3d), for 14 d. Under these conditions, both NMN and NR significantly upregulated meibomian 3β-HSD activity (right versus left eye; Fig. 4e). These improvements in 3β-HSD activity were accompanied by increases in BrdU-incorporated proliferative acinar cells (Fig. 4f and Extended Data Fig. 4b). NMN treatment was not effective for *Hsd3b6*^−/−^ mice; thus, 3β-HSD is essential for supplemental NMN action (Fig. 4f, *Hsd3b6*^−/−^). Crucially, night-time eye-drop administration of NMN was less effective than the daytime regimen in causing increase in BrdU-labeled cells (Fig. 4g,h). With these observations, a longer-term instillation was performed, wherein 21-month-old mice received NMN or NR four times per day during daytime for 90 d (Fig. 4i). Whole-mount lipid staining revealed meibomian gland atrophy in placebo-treated mice. In contrast, mice treated with NMN or NR had significantly enlarged meibomian glands in the right eye, approximately 1.14-fold on average, relative to those in the left eye (*P* < 0.05, for both NMN and NR), which approaches ~30% recovery to the level of 6 months old (Figs. 1d and 4i). Consequently, EDE signs were ameliorated by NMN or NR treatment compared to those with vehicle treatment (Fig. 4j), demonstrating that both NMN and NR can reduce meibomian gland atrophy and associated dry eye disease.

**Extended Data Fig. 5:**
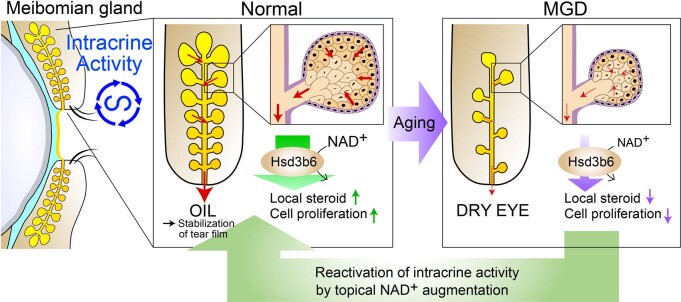
Schematic representation of the potential therapeutic utility of reactivation of local intracrine activity for treatment of age-associated MGD. Normal 3β-HSD activity ensures the high turnover rate required by the meibomian gland to sustain its homeostasis. Aging attenuates meibomian NAD^+^-dependent steroidogenic activity, leading to MGD and MGD-associated dry eye disease. NAD^+^ precursor instillation on eyes restores NAD^+^-dependent enzymatic activity of 3β-HSD, which in turn recovers meibomian gland acinar cell proliferation and mitigates MGD. Hsd3b6 and its human counterpart HSD3B1 are the isozyme responsible for the meibomian gland tissue local 3β-HSD activity.

Topical administration of NMN increased NAD^+^ levels in the meibomian gland (Fig. 4k). The NAD^+^ levels in the meibomian gland significantly increased within 5 min post-administration, peaking at 1 h and returned to basal levels by 2.5 h. Topical administration to the right eye had little effect on NAD^+^ levels in the contralateral left eye, the kidney or the liver of the same animal (Fig. 4k and Extended Data Fig. 4c). A similar NAD^+^ response was also observed 1 h after single NR administration. Repeated topical administration of NMN or NR for 14 d to 18-month-old mice increased the expression of the meibomian morphogenic factors, *Wnt2* and *Adamts17*, as observed in the FACS-purified cells (Fig. 4l). Our data therefore clarified that eye-drop administration of NAD^+^ precursors can lead to reactivation of local meibomian gland activity at the coenzyme, enzyme, downstream gene, cell proliferation, morphology and consequent EDE-protective output level. Based on these data, we conclude that maintenance of local intracrine activity is critical to restore tissue homeostasis of the meibomian gland (Extended Data Fig. 5, model).

## Discussion

Aging is associated with the loss of sex-steroid hormones in men (andropause) and women (menopause). These hormones exert pleiotropic effects; therefore, their systemic application (or exogenous hormone replacement therapy) is limited, owing to their adverse side effects^26,27^. The potential merit of topical (re)activation of local intracrine activity, particularly for the treatment of age-associated MGD and EDE disease, warrants further clinical evaluation because nonsystemic application might not only reduce side effects but also increase drug efficacy. We used an NAD^+^ precursor (nonsteroidal agent), instead of the steroid hormone per se, which allows further alleviation of side effects^28^. In this respect, it is worth noting that NAD^+^ repletion leads to reactivation of innate intracrine activity, allowing steroid synthesis to occur where required (Extended Data Fig. 5).

Our study revealed the presence of intracrine activity in the meibomian gland and defined the extent of its impact on physiology through deletion of the meibomian 3β-HSD activity. This enzyme is responsible for approximately 50% of the local tissue testosterone (the remainder is from circulation) and its absence leads to severe atrophy of the meibomian gland. We showed that this enzyme activity declines with age but can be reactivated through eye-drop NAD^+^ precursor application. NAD^+^ functions as a coenzyme of 3β-HSD, but alternative modes of action might be simultaneously possible. The direct deacetylation of Hsd3b6/HSD3B1 by sirtuin, which requires NAD^+^ to exert its deacetylase activity and is involved in aging^25^, is a potential mechanism that requires further investigation. NAD^+^ also plays a beneficial role in circadian clock reprogramming^29^, which might indirectly improve circadian 3β-HSD activity rhythm. Our results do not exclude the possibility that NAD^+^ influences the other cell types, such as the cornea, conjunctiva and other related cells^30,31^.

We chose the eye-drop approach, rather than oral/intravital injection, because this approach is topical and has fewer effects on distal tissues. Topically administered drugs reach the meibomian gland mainly through the conjunctiva^32^. We administered drugs during the daytime, the time for circadian activation of endogenous 3β-HSD activity. In both humans and mice, the activity of the meibomian gland is considered to increase during rest or sleep when parasympathetic system is dominant^4,33^. This may relate to the observed daytime-better-than-night-time efficacy in mice. Because mice are nocturnal, daytime dosing cannot be guaranteed through dietary supplementation that relies on spontaneous feeding behavior. Whether a non-eye-drop approach could be used to improve local meibomian gland tissue function requires further investigation. It has been reported that NMN, either given intraperitoneally or through drinking water, has an effect on the function of the photoreceptors and lacrimal gland^30,31^.

In both male and female, a peculiar but evolutionarily conserved subtype of 3β-HSD is abundantly expressed in the meibomian gland. In human females, all androgens are essentially elaborated in peripheral tissues from DHEA which declines with age^2^. In males androgen deficiency through antiandrogen therapy has been associated with reduced quantity and quality of meibomian gland secretion^34,35^, suggesting the potential of androgen treatment. Our data only provided evidence for quantitative increase of the meibomian gland by intracrine activity; its connection to quality will need to be studied. The current treatment options for MGD are primarily palliative and limited to eyelid hygiene with warm compression and empirical antimicrobial therapy^5,28^. Reactivation of local intracrine activity via NAD^+^-dependent 3β-HSD activity could provide a mechanism-based approach for the treatment of age-associated MGD and dry eye disease.

## Methods

### Meibography

Meibography was performed at the Kyoto City Hospital, which is affiliated with the Kyoto Prefectural University of Medicine (KPUM), Japan. This study was approved by the Institutional Review Boards of Kyoto City Hospital and KPUM and performed in accordance with the principles of the Declaration of Helsinki. Written informed consent was obtained from all volunteers in this study. Twelve healthy, non-dry eye/non-MGD volunteers were involved in this study; three young women (aged 31.7 ± 2.3 (mean ± s.d.) years), three young men (32.6 ± 3.2 years), three elderly women (63.7 ± 3.1 years) and three elderly men (66.0 ± 4.0 years); all participants were native Japanese. The following participants were excluded from the study; tobacco smokers, contact lens wearers and individuals with any eye and/or systemic disease or who were taking medication at the time of the study. Non-dry eye/non-MGD was diagnosed according to each of the Japanese diagnostic criteria^36^. The meibomian gland images from both upper and lower eyelids were obtained using noncontact meibography (TOPCON).

### Human eyelid specimens

A formalin‐fixed, paraffin‐embedded human eyelid sample obtained from a postmortem 53-year-old male donor who had no history of eye-related diseases was obtained from the Department of Frontier Medical Science and Technology for Ophthalmology at the KPUM; this study protocol was approved by the Institutional Review Boards of both KPUM and Kyoto University. A formalin‐fixed eyelid tissue sample from a postmortem 34-year-old female donor who had no history of eye-related diseases was purchased from Science Care.

### Animals

Young and aged C57BL/6J male mice were purchased from CREA Japan. The *Hsd3b6*^fxneo/+^ mouse was generated at RIKEN (accession no. CDB1067K; http://www2.clst.riken.jp/arg/micelist.html). *CAG-FLPe* (accession no. RBRC01834) and *CAG-Cre* mice (accession no. RBRC01828) were obtained from RIKEN to generate *Hsd3b6*^fl/fl^ and *Hsd3b6-*null (*Hsd3b6*^−/−^) mice, respectively. *Bmal1-*null (*Bmal1*^−/−^) mice^37^, *PER2::LUC* mice^18^ and *K14-Cre* mice^11^ were bred as described^19,38^. As for *Bmal1*^−/−^ mice, we used 9-week-old mice, which had not started to develop a premature aging phenotype or manifest ocular abnormalities^39,40^. *Hsd3b6*^ΔMG^ mice were generated by crossing *Hsd3b6*^fl/fl^ and *K14-Cre* mice. Currently, there is no specific Cre line for meibomian gland expression; ontogenically, the meibomian gland and skin sebaceous gland are closely related, with no selective biomarker and both expressing Hsd3b6 (ref. ^21^). Therefore, we focused on differentiating between the meibomian sebaceous gland and other major circulatory organs, such as gonads and adrenal gland. Mice were housed under a regular 12-h light/12-h dark cycle (lights on at 8:00 and off at 20:00) with free access to food and water. All mouse experiments were conducted in compliance with the Ethical Regulations of Kyoto University and performed under protocols approved by the Animal Care and Experimentation Committee of Kyoto University and Institutional Animal Care and Use Committee of RIKEN Kobe Branch.

### Paraffin section immunohistochemistry

Four-µm-thick eyelid paraffin sections were antigen-retrieved by pressure cooking in Tris-EDTA buffer (pH 9.0) as described^21^ and immersed into PBS containing 0.1% Tween-20. Sections were blocked with 5% FBS and 5% BSA in PBS for 1 h and incubated with a specific set of antibodies, including anti-HSD3B1 (mouse monoclonal, 3C11-D4, Abnova, final 0.05 µg ml^−1^)^8^, anti-Hsd3b6 (rabbit polyclonal^21^, 0.6 µg ml^−1^), anti-Per2 antibody (rabbit polyclonal, PER21-A, Alpha Diagnostic, 1:500 dilution), anti-Luc (mouse monoclonal, NB600-307, Novus, 1:500 dilution) and anti-Itgav (rabbit monoclonal, ab179475, Abcam, 1:300 dilution) for 24 h at 4 °C. The immunoreactivities were visualized with 3,3-diaminobenzidine (brown staining for HSD3B1 and Hsd3b6) and/or ImmPACT Vector Red (Vector Labs for Luc) using horseradish peroxidase-labeled anti-IgG polymers (Dako, EnVision^+^ System-HRP Labeled Polymer anti-mouse for HSD3B1 and anti-rabbit for Hsd3b6) and alkaline phosphatase-based anti-mouse IgG polymers (Nichirei, Histofine simple stain kit AP (M)) or visualized using Alexa594-conjugated anti-rabbit IgG (Thermo Fisher Scientific, 1:1,000 dilution for Per2 and Itgav). Where specified, sections were counterstained with hematoxylin. For immunofluorescence, sections were mounted in medium containing 4ʹ,6-diamidino-2-phenylindole (DAPI) for counterstaining cell nuclei. For the experiment examining the expression of PER2::LUC and Hsd3b6, tissue sections were prepared from mice at the peak time point of the Per2 expression in the meibomian gland (CT20; Supplementary Fig. 5).

### Whole-mount meibomian gland visualization

The area of the meibomian gland, cut in cross section, can be variable depending on the position of the section. We therefore developed a whole-mount histology method for accurate measurement of the area of the meibomian gland. The skin around the eye, containing both upper and lower eyelids, was excised as a whole tissue. After fixation with 4% paraformaldehyde, the tissues were transferred into 50% ethanol for 10 min and 70% ethanol for 20 min, then stained with Sudan IV-saturated 70% ethanol solution containing 2% NaOH for 24 h. Following extensive washes with 70% ethanol, the specimens were cleared by incubation in 25% *N*,*N*,*N*ʹ,*N*ʹ-Tetrakis-(2-hydroxypropyl) ethylenediamine (Tokyo Chemical Industry) in glycerol overnight. Before being mounted in glycerin jelly, the lateral canthi of the eyes were cut open to make the specimens flat and adhering connective tissues and debris were removed. Images of whole-mount meibomian glands were taken under a light-field microscope and the areas of meibomian glands were measured using ImageJ (https://imagej.nih.gov/ij/).

### Imaging mass spectrometry

Imaging MS was performed as described^41^. In brief, frozen eyelid tissue sections (10 μm), mounted on ITO-coated glass slides for MALDI MS imaging, were spray-coated with Girard’s T (Gir-T) reagent. Mass spectra were acquired using a MALDI linear ion trap mass spectrometer (MALDI LTQ XL; Thermo Fisher Scientific) equipped with a 60-Hz N_2_ laser. The laser spot size was approximately 120 μm; each pixel was irradiated 50 times at a repetition rate of 20 Hz. MS was run in the positive ion detection mode. Two specific multiple reaction monitoring (MRM) transitions were monitored for testosterone: *m*/z 402 > 343 > 315 and 402 > 343 > 163. The two spectral datasets were each transformed into image data using ImageQuest software v.1.0.1 (Thermo Fisher Scientific)^41^.

### Quantification of 3β-HSD activity

3β-HSD enzymatic activities were measured as described^10^ using ^3^H-DHEA (PerkinElmer). The tarsal plates were surgically isolated from the upper and lower eyelids. This procedure involved making a small incision near the inner corner of the eyelid, separating skin/subcutaneous tissue from the inner to outer aspect of the lid and then removing skin from the tarsal plates by cutting at the mucocutaneous junction. After these steps, the tarsal plates were cut into slices (0.2 mm thickness) with a tissue chopper (McIlwain laboratory) and transferred into pre-aerated (5% CO_2_ and 95% O_2_) Hank’s balanced salt solution (HBSS) containing ^3^H-DHEA (40 nM), dutasteride (10 µM, Cayman), fadrozole hydrochloride (10 µM, Sigma) and 4% propylene glycol. After 30 min at 37 °C, the medium was collected and immediately extracted into ethyl acetate. The extracts were evaporated to dryness under a nitrogen stream at 75 °C. The dry extracts were reconstituted into 40% acetonitrile and subjected to HPLC-connected RI scintillation as described^10^.

### Quantification of meibomian tissue testosterone

Male mice were castrated or sham-operated at 14 weeks of age and, after a recovery period of 14 d, tarsal plates were isolated. Approximately 2–3 mg of the tarsal plates were homogenized in methanol/H_2_O (3:1) and extracted into dichloromethane using ISOLUTE columns (Biotage). Following evaporation to dryness, this fraction was reconstituted in methanol containing 5% acetic acid and processed for derivatization using the Amplifex Keto Reagent (AB Sciex)^42^. The concentration of the testosterone derivative (MRM transition, *m*/z 403 > 164) was measured using LC–MS/MS QTRAP 4500 system (AB Sciex) and normalized to the weight of the tissue analyzed.

### BrdU incorporation assay

BrdU is a thymidine analog that incorporates into dividing cells during DNA synthesis. Mice were injected intraperitoneally with BrdU (100 mg kg^−1^ body weight) and 30 min later, eyelids were collected and fixed with 4% paraformaldehyde. Paraffin section immunohistochemistry was performed using anti-BrdU antibody (Rockland, 600-401-C29). The perimeter of the meibomian gland acini was measured using ImageJ. The total number of BrdU^+^ cells (per perimeter) was counted using at least 20 different sagittal sections obtained from the central region of the lower eyelid.

### EDE test

Mice were anesthetized intraperitoneally with ketamine (50 mg kg^−1^) and xylazine (20 mg kg^−1^) just before the experiment and placed in a constant air humidity of around 15% for 1 h. After this imposed desiccation, 10 µl of 1% sodium fluorescein (Fluorescite, Novartis Pharma) was instilled onto the ocular surface. Corneas were rinsed with saline and photographed with a slit lamp biomicroscope (SL130; Carl Zeiss) under cobalt blue light 2 min after fluorescein instillation. Corneal punctate staining areas were scored in a blinded fashion with a grading system of 0 to 4 as follows: grade 0, absent; grade 1, about 12.5% staining of the ocular surface; grade 2, about 25% staining of the ocular surface; grade 3, about 50% staining of the ocular surface; and grade 4, about 100% staining of the ocular surface. Corneal epithelial staining was performed 3 d before the above assay (baseline) using the same protocol except that mice were subjected to staining soon after anesthesia. Lacrimal gland excision was performed as described^43^. An incision was made in the temporal area, approximately 3 mm to the temporal side of the eye, anterior to the ear. The exorbital lacrimal gland was exposed and excised using ophthalmic forceps under a stereomicroscope. The lacrimal excision was performed bilaterally. The incisions were sutured and antibiotic ointment was applied^43^. Before and 4, 8 and 15 d after surgery, corneal epithelial staining was performed without imposed desiccation.

### Assessment of tear volume

Tear volume was assessed using phenol red impregnated Zone-Quick cotton threads (Ayumi Pharmaceutical). The threads were held with jeweler forceps and applied in the ocular surface in the lateral canthus for 60 s. Wetting of the thread was measured in mm, using the scale on the cotton thread.

### FACS purification of meibomian gland cells

Tarsal plates from four mice per replicate were pooled in HBSS containing 0.25% trypsin and 50 µg ml^−1^ DNaseI and incubated at 37 °C for 15 min. After mincing, tissues were dissociated in PBS containing 20% FBS and 0.5 mM EDTA and filtered through a 70-µm strainer. Cell were resuspended into ice-cold PBS containing 2% FBS, 0.5 mM EDTA and 50 µg ml^−1^ BSA and incubated with anti‐CD45-APC/cyanine7 (BioLegend, 103116, 1:100 dilution) and anti‐Itgav-PE (BioLegend, 104106, 1:100 dilution) for 1 h. Cell sorting was performed using a FACSAria II Flow cytometer (BD Biosciences). Itgav^+^;CD45^−^ meibomian gland cells (approximately 10,000 cells per replicate) were sorted into DMEM/Ham’s F12 medium containing 10% FBS. After sorting, cells were pelleted and total RNA was directly extracted using an RNeasy micro kit (QIAGEN). In our study, CD45 expression was used to exclude hematopoietic cells from the analysis.

### RNA-seq

Total RNA was amplified using a SMARTer Ultra Low Input RNA kit for sequencing (Clontech). RNA-seq libraries were prepared using a NEBNext Ultra II RNA Library Prep kit for Illumina (NEB) according to the manufacturers’ protocols. Libraries were single-end-sequenced on a HiSeq 1500 sequencer (Illumina). Reads were aligned to the mm10 mouse genome using STAR aligner^44^. Expression analysis of the RNA-seq data was performed using HOMER^45^. Differentially expressed genes were analyzed using DESeq2 (ref. ^46^).

GO enrichment was calculated with GOrilla online tool (http://cbl-gorilla.cs.technion.ac.il/). The complete list of enriched GO terms is available in Supplementary Data 1.

### Laser microdissection

Cryosections (20-μm thick) of fresh-frozen eyelids were mounted on a POL-membrane slide (Leica) as described^10^. For RNA analysis, sections were fixed in ice-cold ethanol-acetic acid mixture (19:1) for 2 min and stained with 0.05% toluidine blue. Meibomian glands were then excised using a LMD7000 device (Leica) and lysed into Trizol reagent (Invitrogen). For the measurement of NAD^+^ concentration, sections were fixed in ice-cold 50% ethanol for 30 s and meibomian glands were selectively excised into 50% methanol. The total size of the areas of the meibomian glands that were captured by laser microdissection was quantified for each sample using the Leica LMD software (Leica) for data normalization.

### Quantitative RT–PCR

RNA was extracted using RNeasy micro kit (QIAGEN) and converted to complementary DNA with SuperScript VILO cDNA synthesis kit (Thermo Fisher Scientific). Quantitative real-time PCR was achieved either using THUNDERBIRD SYBR qPCR Mix (TOYOBO) with StepOnePlus system (Applied Biosystems) or by TaqMan qPCR analysis performed on a BioMark HD System (Fluidigm) using a 48.48 Fluidigm BioMark Dynamic Array chip (Fluidigm) as described previously^19,38^. For data collection and analysis, StepOne Software v.2.2.2 (Applied Biosystems) and Fluidigm Real-Time PCR Analysis v.4.1.3 (Fluidigm) were used.

### PER2::LUC bioluminescence monitoring

The eyelids from *Per2*^Luc^ mice were cut into slices (300 μm thickness) with a tissue chopper (McIlwain laboratory) and placed on a Millicell membrane (PICMORG50, Millipore) with a CO_2_-independent DMEM containing 10 mM HEPES (pH 7.2), 2% B27 (Thermo Fisher Scientific) and 1 mM d-luciferin (Promega) at 35 °C. Bioluminescence was continuously monitored without interruption for >5 d with a highly sensitive CCD camera (600 series, Spectral Instruments) as described^47^. Time-lapse images were acquired every 30 min with exposure time of 29 min and luminescence intensity of the area of interest was measured using ImageJ. For Nile red lipid staining, we used an LV200 imaging system (Olympus). After bioluminescence imaging, the slice on the membrane was dipped in PBS containing 1 μg ml^−1^ Nile red (MP Biomedicals) for 45 min. Then, fluorescence was subsequently imaged using the same LV200 system. LV200 imaging analysis was performed with Molecular Devices MetaMorph v.7.8.9.0.

### Western blot

The antibody that we developed for Hsd3b6 (ref. ^21^) could not be used for SDS–PAGE-based blotting^21^. We thus employed Coomassie brilliant blue-based blue native PAGE for immunodetection of Hsd3b6. Meibomian glands were homogenized in 50 mM Tris-HCl (pH 7.2) buffer containing 1 mM dithiothreitol and 1× cOmplete Protease inhibitor cocktail (Roche diagnostics), followed by centrifugation at 106,000*g*. The pellet was resuspended in NativePAGE Sample Buffer (Invitrogen) containing 1 mM dithiothreitol and 1% digitonin and mixed with NativePAGE 5% G-250 Sample Additive (Invitrogen) before loading on the NativePAGE Novex Bis-Tris Gel (Invitrogen). Electrophoresis was performed at a constant current of 1 mA at 4 °C. Proteins in the gel were electroblotted to a polyvinylidene difluoride membrane with a buffer containing 25 mM Tris, 200 mM glycine, 10% methanol and NativePAGE Cathode Additive (Invitrogen) at a constant current of 350 mA for 1.5 h. Membranes were incubated further with PBS containing 0.05% Tween-20 for 24 h to remove Coomassie brilliant blue on the blots. After incubation with a Blocking One buffer (Nacalai), blots were probed for Hsd3b6 (anti-Hsd3b6) and its immunoreactivities were visualized using chemiluminescence. Immunoblot for Per2 was performed as described^38^. Full-length western blots are presented in Supplementary Information.

### Quantification of NAD^+^ in the meibomian gland

Concentrations of NAD^+^ were determined by LC–MS/MS, using a Shimadzu Nexera UHPLC/HPLC System (Shimadzu) coupled to a LC–MS-8040 triple quadrupole mass spectrometer (Shimadzu). Meibomian gland cells, selectively isolated into 50% methanol (see above), were sonicated with chloroform (1:1). After centrifugation, supernatants were filtered (0.22 µm) and freeze-dried. The samples were resolved in water before analysis and separated through a COSMOSIL Packed Column 5C_18_-PAQ (2.0 mm × 150 mm, Nacalai). NAD^+^ was detected using the multiple reaction monitoring mode and identified by comparison of its LC retention time and MS^2^ fragmentation pattern (*m*/*z* 663.85 > 136.05) with those of the authentic standard. NAD^+^ levels were quantified based on the peak area compared to a standard curve. The values of NAD^+^ were normalized to the tissue volume (mm^3^) captured by laser microdissection.

### In vitro 3β-HSD activity assay with different doses of NAD^+^

The tarsal plates were homogenized in PBS containing 1× cOmplete Protease inhibitor (Roche Diagnostics). After brief centrifugation at 700*g*, the supernatant was centrifuged at 106,000*g* for 1 h at 4 °C. Then, the pellet (microsomal fraction) was resuspended in PBS containing 1× cOmplete Protease inhibitor cocktail and its protein content was determined with a Bradford assay. Approximately 5 µg of purified microsomal protein was incubated in vitro in a reaction buffer (10 mM phosphate, pH 7.5, 140 mM NaCl and 4% propylene glycol) containing 100 nM ^3^H-DHEA and a different dose of NAD^+^. After a 1-h incubation at 37 °C, the reaction was stopped by adding ethyl acetate. The steroids were extracted from the sample and subjected to HPLC as described^10^.

### Pharmacological treatment of H295R cells

H295R cells (ATCC, CRL-2128), which express endogenously HSD3B1 (ref. ^48^), were cultured as described^48^ in DMEM/F12 supplemented with 2.5% Nu serum (BD Biosciences) and 1% ITS premix (BD Biosciences). Cells were cultured in a 35-mm dish with either only FK866 (10 nM) or in combination with NMN or NR (100 µM) for 24 h and treated with ^3^H-DHEA for an additional 1 h. The steroids were extracted from the medium and subjected to HPLC as described previously^10^.

### Quantification of NAD^+^ in H295R cells by LC–UV

NAD^+^ levels in H295R cells were quantified as described^49^ by LC–UV, using an HPLC instrument (Shimadzu, LC-20AT) equipped with a UV detector (Shimadzu, SPD-20A). Cells were lysed into 10% perchloric acid and neutralized with 3 M K_2_CO_3_. After centrifugation, extracts were filtered (0.22 µm) and loaded on a column (COSMOSIL 5C_18_-PAQ, 4.6 mm × 150 mm). Analysis was run at a flow rate of 1 ml min^−1^ with 100% buffer A (0.05 M phosphate buffer, pH 7.0) from 0 to 3 min, a linear gradient to 95% buffer A/5% buffer B (methanol) from 3 to 4 min, 95% buffer A/5% buffer B from 4 to 9 min, a linear gradient to 85% buffer A/15% buffer B from 9 to 11 min, 85% buffer A/15% buffer B from 11 to 21 min and a linear gradient to 100% buffer A from 21 to 22 min. NAD^+^ levels were monitored by absorbance at 261 nm. The values were quantified based on the peak area compared to a standard curve^49^. The limit of detection of NAD^+^ was approximately 0.1 µM in this assay. Data were normalized to the number of cells analyzed.

### Eye-drop administration

PBS containing either 5% NMN or NR was applied onto the right eye six times a day at 9:00, 11:00, 13:00, 15:00, 17:00 and 19:00 for 2 weeks (5 µl of the drug solution was administered at each time point). The left eye was not treated for control. To investigate the effect on the morphology of the meibomian gland and EDE, the same 5% compound solution was applied four times per day at 9:00, 12:00, 15:00 and 18:00 for 90 d. For night-time administration, eye-drop instillation was performed under dim red light conditions (<2 lux) at 21:00, 0:00, 3:00 and 6:00.

### Statistical analysis

Statistical analyses were performed with GraphPad Prism 8, using statistical tests for each figure as indicated. Circadian oscillations of 3β-HSD enzymatic activity were assessed using the nonparametric JTK-Cycle^50^.

### Reporting Summary

Further information on research design is available in the Nature Research Reporting Summary linked to this article.

## Supplementary information

Supplementary Information Supplementary Figs. 1–6.

Reporting Summary

Supplementary Data 1 RNA-seq data and GO enrichment.

## Data Availability

Data that support the findings of this study are available as Source Data and Supplementary Data. RNA-seq datasets generated in this study are available at Gene Expression Omnibus (accession no. GSE166784).

## Source data

Source Data Fig. 1 Statistical Source Data.
Source Data Fig. 2 Statistical Source Data.
Source Data Fig. 3 Statistical Source Data.
Source Data Fig. 3 Unprocessed western blot data.
Source Data Fig. 4 Statistical Source Data.
Source Data Extended Data Fig. 1 Statistical Source Data.
Source Data Extended Data Fig. 2 Statistical Source Data.
Source Data Extended Data Fig. 3 Statistical Source Data.
Source Data Extended Data Fig. 4 Statistical Source Data.
