# Supplementary material for: Intracrine activity involving NAD-dependent circadian steroidogenic activity governs age-associated meibomian gland dysfunction
Source: Nat Aging. 2022 Feb 10;2(2):105–14. doi: 10.1038/s43587-021-00167-8 (PMC10154200; doi:10.1038/s43587-021-00167-8)
Supplement: Supplementary file 1 — Supplementary Information Supplementary Figs. 1–6. [file 43587_2021_167_MOESM1_ESM.pdf]

nature portfolio

https://doi.org/10.1038/s43587-021-00167-8

Supplementary information

Intracrine activity involving NAD-dependent circadian steroidogenic activity governs age-associated meibomian gland dysfunction

In the format provided by the authors and unedited

Supplementary Information for

Intracrine activity involving NAD-dependent circadian steroidogenic activity governs age-associated meibomian gland dysfunction

Lena Sasaki, Yuki Hamada, Daisuke Yarimizu, Tomo Suzuki, Hiroki Nakamura, Aya Shimada, Khanh Tien Nguyen Pham, Xinyan Shao, Koki Yamamura, Tsutomu Inatomi, Hironobu Morinaga, Emi K. Nishimura, Fujimi Kudo, Ichiro Manabe, Shogo Haraguchi, Yuki Sugiura, Makoto Suematsu, Shigeru Kinoshita, Mamiko Machida, Takeshi Nakajima, Hiroshi Kiyonari, Hitoshi Okamura*, Yoshiaki Yamaguchi, Takahito Miyake, Masao Doi*

*Address correspondence to:
Masao Doi, doimasao@pharm.kyoto-u.ac.jp
Hitoshi Okamura, okamura.hitoshi.4u@kyoto-u.ac.jp

This PDF file includes:

Supplementary Figs. 1 to 6

Supplementary Fig. 1

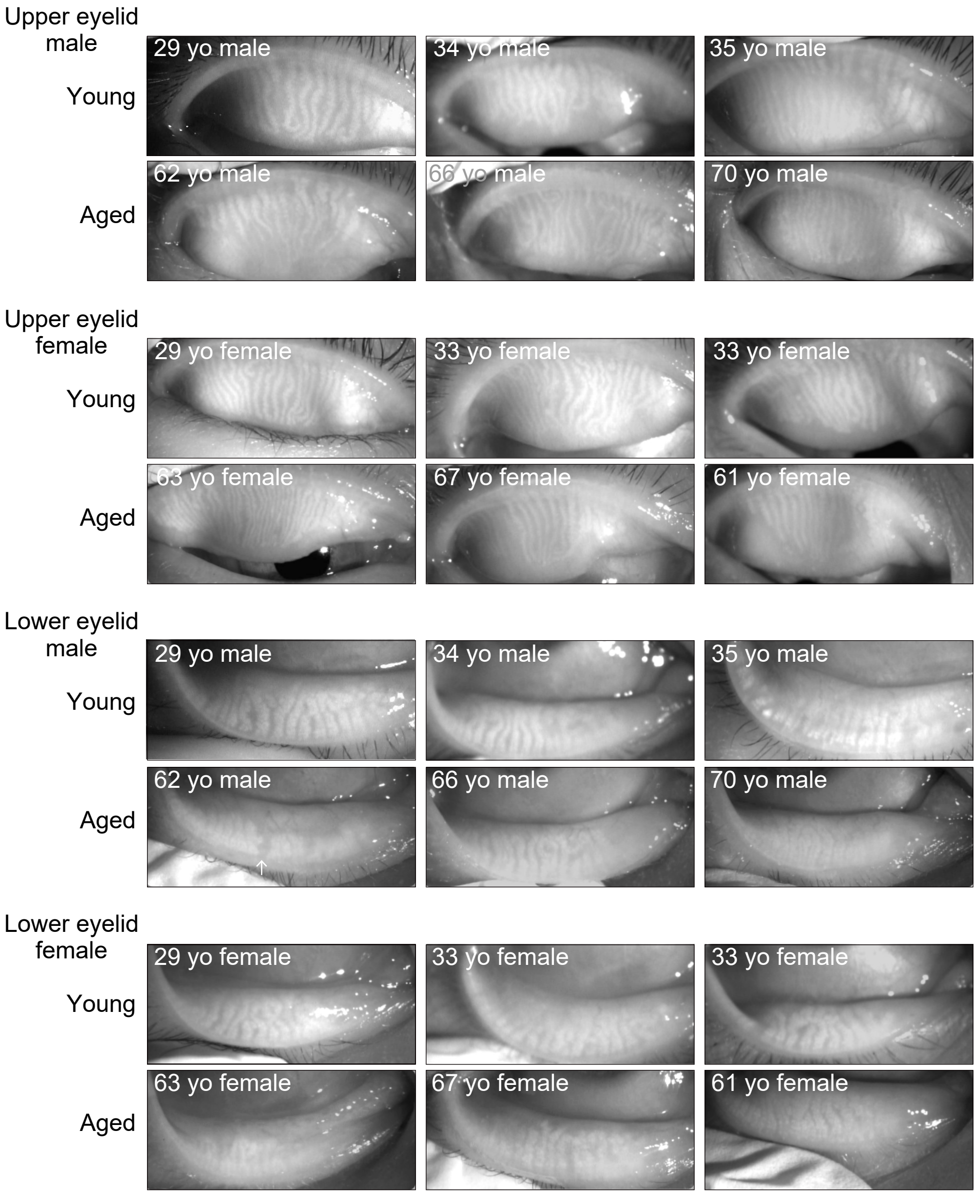

Supplementary Fig. 1 | Meibography images of upper and lower eyelids of young and aged men and women, analyzed in Fig. 1a. Arrow, the area of the dropout. Meibomian gland dropout refers to the loss of acinar tissue detected meibography.

Supplementary Fig. 2

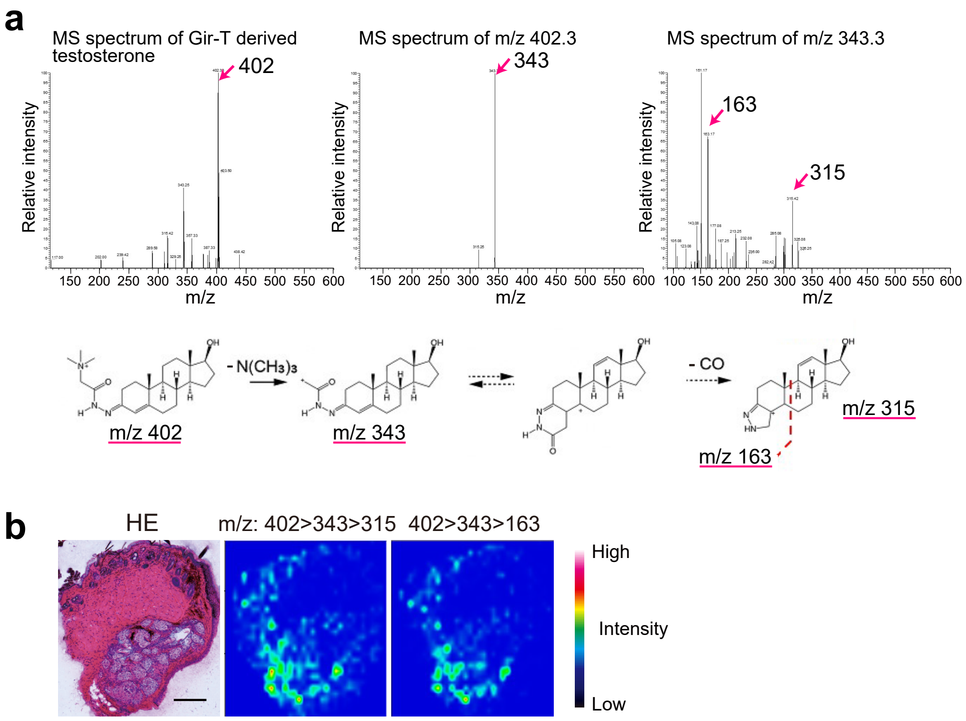

Supplementary Fig. 2 | Imaging mass spectrometry of testosterone in a mouse sagittal eyelid section, related to Fig. 1e. a, Product ion sepctra of GirT-testosterone, obtained by MS, MS^2, and MS^3. Proposed fragmentation pathway of GirT-testosterone is shown below. **b**, Distribution of o GirT-testosterone derived product ions (m/z 315 and 163) in the mouse eyelid as determined by MALDI-imaging mass spectrometry. The section was counterstained with hematoxylin and eosin (HE). Scale bar, 200 µm. Data are representative of four biologically independent eyelid samples with similar results.

Supplementary Fig. 3

a

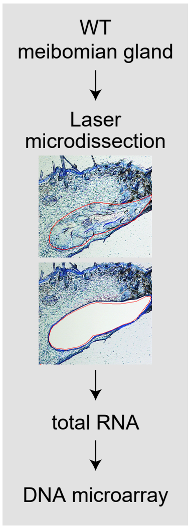

Integrin family

Gene symbol	Microarray intensity	Probe ID	Gene full name
Itgav	637.8	17372515	Integrin alpha V
Itfg1	583.6	17511389	Integrin alpha FG-GAP repeat containing 1
Itgb1	510.6	17507014	Integrin beta 1
Itga6	424.1	17371800	Integrin alpha 6
Itgb5	354.9	17325109	Integrin beta 5
Itgb8	263.7	17284839	Integrin beta 8
Itgb2	194.8	17234647	Integrin beta 2
Itgax	122.1	17483615	Integrin alpha X
Itga2	119.7	17296355	Integrin alpha 2
Itga1	119.2	17296388	Integrin alpha 1
Itgam	105.2	17483577	Integrin alpha M
Itga8	100.4	17381717	Integrin alpha 8
Itgb4	97.5	17258473	Integrin beta 4
Itga5	84.0	17322369	Integrin alpha 5
Itfg2	80.6	17471324	Integrin alpha FG-GAP repeat containing 2
Itgal	65.7	17483264	Integrin alpha L
Itgae	64.9	17252574	Integrin alpha E, epithelial-associated
Itga4	61.2	17372307	Integrin alpha 4
Itga3	60.3	17268137	Integrin alpha 3
Itgb3	57.6	17257235	Integrin beta 3
Itgb6	54.9	17385654	Integrin beta 6
Itga7	52.1	17238605	Integrin alpha 7
Itgb7	49.6	17322163	Integrin beta 7
Itga11	46.1	17518191	Integrin alpha 11
Itga2b	45.5	17270222	Integrin alpha 2b
Itga9	37.7	17522916	Integrin alpha 9
Itgb2l	10.4	17332473	Integrin beta 2-like

b

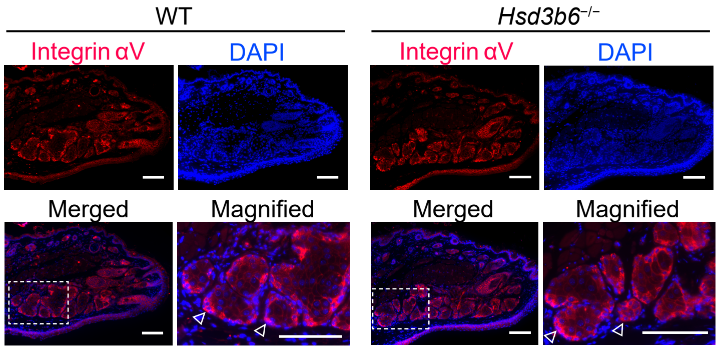

c

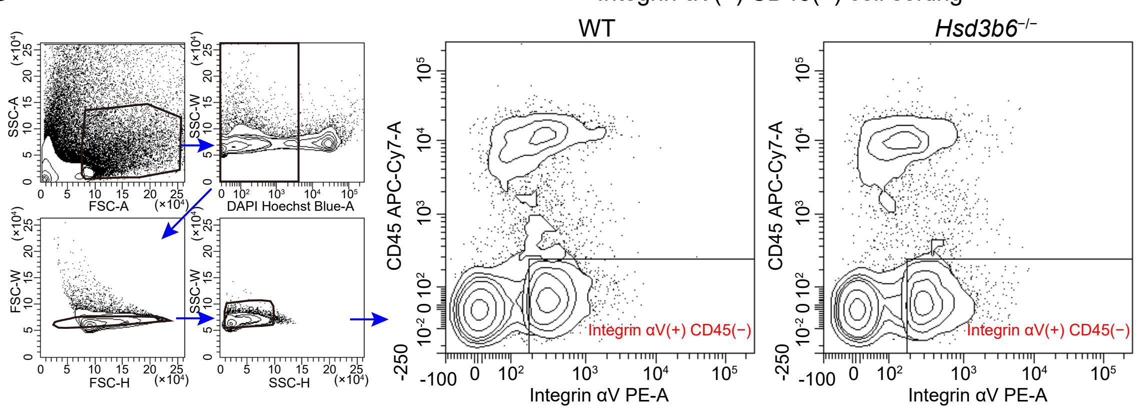

Supplementary Fig. 3 | Cell sorting of Integrin αV (Itgav)-positive meibomian gland cells from WT and *Hsd3b6*$^{-/-}$ mice, related to Fig. 2k. **a**, **b**, Identification of Itgav as a dominant Integrin subtype in the meibomian gland. **a**, Meibomian gland DNA microarray analysis focusing on the Integrin family genes. Note that Itgav was the most abundant subtype in the meibomian gland. **b**, Representative immunofluorescence images of Itgav in WT and *Hsd3b6*$^{-/-}$ eyelid sagittal section. Nuclei were stained with DAPI. The merged image is a combined image of Itgav (red) and DAPI (blue). The boxes indicate the regions enlarged in the bottom right panels. Bars, 100 μm. Note that intense immunopositive signals were observed in the basal acinar cells, i.e., the cells localized in the basal layer of the acini. Arrowheads indicate representative basal acinar cells. In addition, relatively weak but appreciable immunostaining signals were also observed for the cells inside the acini, which perhaps include both differentiating meibocytes and, at least partly, their matured cells. Similar observations were obtained in three biologically independent animals. **c**, Representative FACS plots of Itgav(+);CD45(−) meibomian gland cells from WT and *Hsd3b6*$^{-/-}$ mice ($n=4$ mice for each experiment). In both genotypes, approximately 10,000 cells were collected for the subsequent RNA-seq analysis. Left panels indicate representative FACS plots showing the gating strategy to eliminate debris, dead cells and doublet cells.

Supplementary Fig. 4

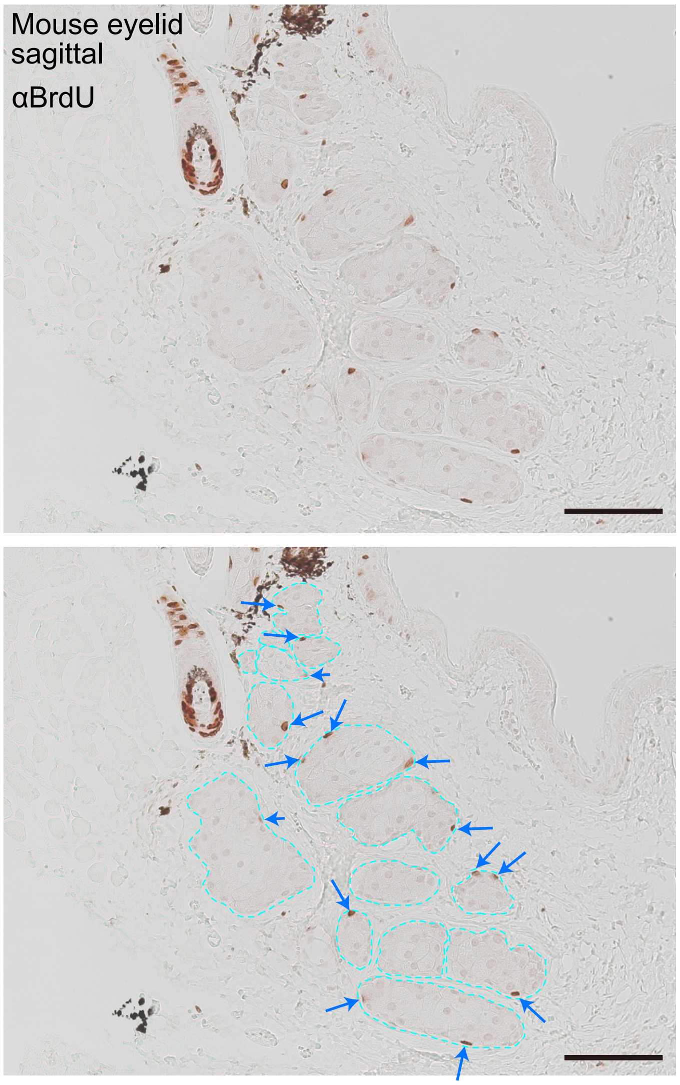

Supplementary Fig. 4 | Representative immunohistochemistry of BrdU incorporation assay, related to Fig. 2n. Mice were injected intraperitoneally with BrdU (100 mg/kg body weight), and 30 min later, tissues were collected for immunohistochemistry with anti-BrdU antibody. Two identical pictures are shown with or without anatomical labeling. Dashed lines indicate representative meibomian gland acini. Arrows indicate BrdU-immunolabeled basal acinar cells. Scale bars, 100 µm. BrdU is a thymidine analog that incorporates into dividing cells during DNA synthesis. Note that actively dividing cells are mainly localized in the basal layer of the meibomian gland acini. This is consistent with the holocrine activity of the gland. Data are representative of biologically independent eyelid samples with similar results.

Supplementary Fig. 5

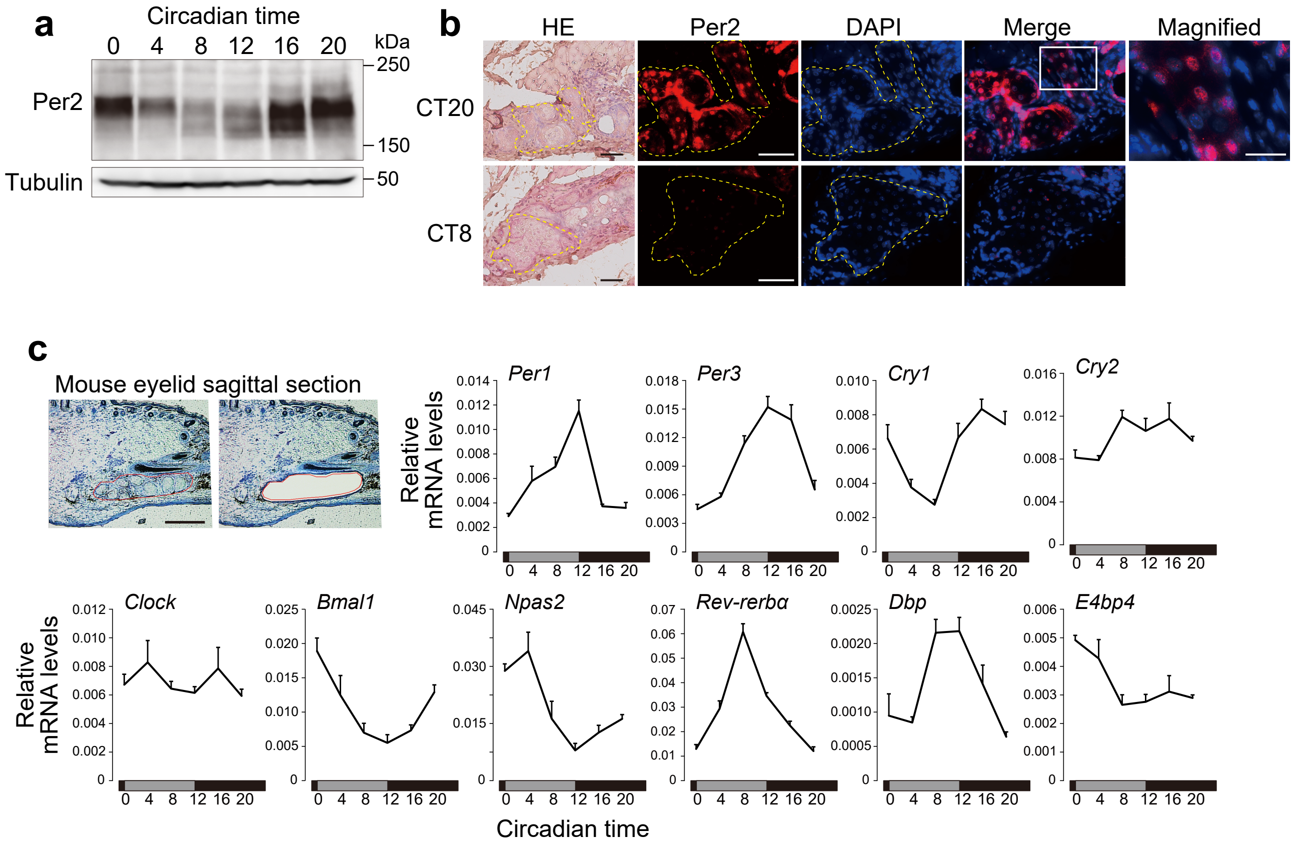

Supplementary Fig. 5 | Circadian profile of the meibomian gland, related to Fig. 3a-c and f. a, Immunoblots showing circadian expression of Per2 protein in the meibomian gland. α-Tubulin was used as a loading control. Tissues were collected from animals in darkness (i.e., without photic time cue) at 4-h intervals starting at CT0 (the onset of the subjective day). **b**, Representative immunofluorescense images of Per2 (red) and DAPI-labeled nuclear staining (blue) in the meibomian glands at the Per2 protein circadian peak (CT20, upper panels) and trough (CT8, lower panels) timepoints. The sections were immunolabeled with anti-Per2 antibody. Merge shows combined images for Per2 and DAPI. The sections were counterstained with hematoxylin and eosin (HE). Dashed lines indicate representative meibomian gland acini. White box indicates area of higher magnification view. Scale bars, 50 µm and 20 µm. **c**, 24-h mRNA expression profiles of circadian clock genes *Per1*, *Per3*, *Cry1*, *Cry2*, *Clock*, *Bmal1*, *Npas2*, *Rev-erbα*, *Dbp*, and *E4bp4* in laser-microdissected meibomian glands. Values at 4-h intervals along the circadian cycle (means ± SEM of $n = 4$ biologically independent mice per time point) were determined by qRT-PCR and normalized to those of *Rplp0*. The micrographs show a representative tissue section stained with toluidine blue pre and post laser microdissection, reproduced from Fig. 3f. Scale bar, 200 µm. Data in **a-b** are representative of four independent experiments with similar results. Full-length western blots in **a** are presented in Supplementary Fig. 6.

Supplementary Fig. 6

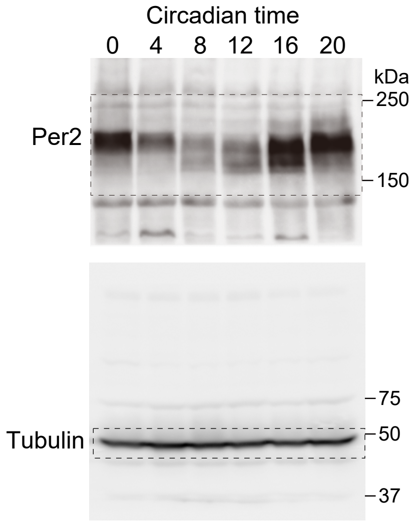

Supplementary Fig. 6 | Uncropped immunoblot images of Supplementary Fig. 5a.
