# Supplementary material for: Intracrine activity involving NAD-dependent circadian steroidogenic activity governs age-associated meibomian gland dysfunction
Source: Nat Aging. 2022 Feb 10;2(2):105–14. doi: 10.1038/s43587-021-00167-8 (PMC10154200; doi:10.1038/s43587-021-00167-8)
Supplement: Supplementary file 2 — Reporting Summary [file 43587_2021_167_MOESM2_ESM.pdf]

nature research

Corresponding author(s): Masao Doi

Last updated by author(s): Nov 22, 2021

Reporting Summary

Nature Research wishes to improve the reproducibility of the work that we publish. This form provides structure for consistency and transparency in reporting. For further information on Nature Research policies, see our Editorial Policies and the Editorial Policy Checklist.

Statistics

For all statistical analyses, confirm that the following items are present in the figure legend, table legend, main text, or Methods section.

n/a	Confirmed	
☐	☒	The exact sample size (*n*) for each experimental group/condition, given as a discrete number and unit of measurement
☐	☒	A statement on whether measurements were taken from distinct samples or whether the same sample was measured repeatedly
☐	☒	The statistical test(s) used AND whether they are one- or two-sided<br>*Only common tests should be described solely by name; describe more complex techniques in the Methods section.*
☐	☒	A description of all covariates tested
☐	☒	A description of any assumptions or corrections, such as tests of normality and adjustment for multiple comparisons
☐	☒	A full description of the statistical parameters including central tendency (e.g. means) or other basic estimates (e.g. regression coefficient) AND variation (e.g. standard deviation) or associated estimates of uncertainty (e.g. confidence intervals)
☐	☒	For null hypothesis testing, the test statistic (e.g. *F*, *t*, *r*) with confidence intervals, effect sizes, degrees of freedom and *P* value noted<br>*Give P values as exact values whenever suitable.*
☒	☐	For Bayesian analysis, information on the choice of priors and Markov chain Monte Carlo settings
☒	☐	For hierarchical and complex designs, identification of the appropriate level for tests and full reporting of outcomes
☒	☐	Estimates of effect sizes (e.g. Cohen's *d*, Pearson's *r*), indicating how they were calculated

Our web collection on statistics for biologists contains articles on many of the points above.

Software and code

Policy information about availability of computer code

Data collection	Perkin Elmer ProFSA-Radio HPLC Chromatography v3.4.3.36d, Fujifilm Image Reader LAS-4000 mini v1.0, Thermo Fisher Scientific StepOne Software v2.2.2, Fluidigm Real Time PCR Analysis v4.1.3, Molecular Devices MetaMorph v7.8.9.0, Leica LMD software v6.7.2.
Data analysis	GraphPad Prism 8 and Microsoft office Excel 2019 were used for biostatistical analysis and depicting scientific graphs. NIH Image J 1.53i was used for imaging analysis. Trimmomatic v0.39, STAR v2.7.5a, Homer v4.11, and DESeq2 v1.26.0 were used for RNA-sequencing analysis. FACSDiva v8.0.2 was used for analysis of flow cytometry. ImageQuest software v1.0.1 was used for imaging mass spectrometry.

For manuscripts utilizing custom algorithms or software that are central to the research but not yet described in published literature, software must be made available to editors and reviewers. We strongly encourage code deposition in a community repository (e.g. GitHub). See the Nature Research guidelines for submitting code & software for further information.

Data

Policy information about availability of data

All manuscripts must include a data availability statement. This statement should provide the following information, where applicable:

- Accession codes, unique identifiers, or web links for publicly available datasets
- A list of figures that have associated raw data
- A description of any restrictions on data availability

Data that support the findings of this study are available as Source Data and Supplementary Data. RNA-seq datasets generated in this study are available at Gene Expression Omnibus (accession number: GSE166784).

Field-specific reporting

Please select the one below that is the best fit for your research. If you are not sure, read the appropriate sections before making your selection.

☒ Life sciences ☐ Behavioural & social sciences ☐ Ecological, evolutionary & environmental sciences

For a reference copy of the document with all sections, see nature.com/documents/nr-reporting-summary-flat.pdf

Life sciences study design

All studies must disclose on these points even when the disclosure is negative.

Sample size	All experiments except FACS-based RNA-seq analysis (n=2) were performed with a minimum of 3-4 biological replicates to perform statistical testing and to take into account biological variability. No statistical calculation was performed to predetermine sample size because it was difficult to estimate the extent of biological variation in each case. Nonetheless, the sample size chosen in cell-based studies was sufficient to allow for the determination of statistical significance because we examined cloned cells and/or isolated tissues from inbred animals kept in identical cell culture environments. For animal experiments, cohort size was determined by experimental type, availability of animals, and using standard cohort sizes in our field ranging 4-12 mice/group for in vivo experiments.
Data exclusions	In principle, we did not exclude any data in this study. However, dead mice were excluded from assessment.
Replication	Experiments have been replicated multiple times, using different animal cohorts. The data plotted in figures represent biological replicates. For quantification of NAD+ and gene expression, we analyzed different animals (biological replicates) and made at least two-three technical replicates per animal. For cell line experiments, independent cultures and/or treatments were considered to be biological replicates. For knockout mouse experiments, we used at least two-three different cohorts (different litters). Each cohort include at least 3-4 mice each for sex and genotype. Replicate experiments were successful.
Randomization	Allocation was random for all in vivo animal and in vitro cell culture experiments. Age-matched mice were randomly assigned to control (placebo/sham) or experimental group.
Blinding	BrdU-positive cell counting and evaporative dry eye scoring analysis were performed with the investigator blind to genotype and/or condition. Blinding was not performed for other experiments since measurements were not influenced by observer bias but rather based on quantitative whole-mount imaging analysis, realtime PCR, mass spectrometry, flow cytometry, and scintillation counter detection.

Reporting for specific materials, systems and methods

We require information from authors about some types of materials, experimental systems and methods used in many studies. Here, indicate whether each material, system or method listed is relevant to your study. If you are not sure if a list item applies to your research, read the appropriate section before selecting a response.

Materials & experimental systems

n/a	Involved in the study	
☐	☒	Antibodies
☐	☒	Eukaryotic cell lines
☒	☐	Palaeontology and archaeology
☐	☒	Animals and other organisms
☐	☒	Human research participants
☒	☐	Clinical data
☒	☐	Dual use research of concern

Methods

n/a	Involved in the study	
☒	☐	ChIP-seq
☐	☒	Flow cytometry
☒	☐	MRI-based neuroimaging

Antibodies

Antibodies used	Anti-HSD3B1, mouse monoclonal 3C11-D4, Abnova, H00003283-M01; Anti-Hsd3b6, rabbit polyclonal, home-made; Anti-Per2, rabbit polyclonal, Alpha Diagnostic, PER21-A; Anti-Luc, mouse monoclonal Luci 21 1-107, Novus, NB600-307; Anti-Itgav, rabbit monoclonal EPR16800, Abcam, ab179475; Anti-BrdU, rabbit polyclonal, Rockland, 600-401-C29; Anti-CD45-APC/Cyanine7, rat monoclonal 30-F11, BioLegend, 103116; Anti-Itgav-PE, rat monoclonal RMV-7, BioLegend, 104106; and Alexa594-conjugated anti-rabbit IgG, goat polyclonal, Thermo Fisher Scientific, A-21207.
Validation	Specificity of anti-Hsd3b6 antibody was validated in Fig. 2c. All commercially available antibodies were validated by the manufacturer and validation descriptions are available on the respective website: http://www.abnova.com; https://www.4adi.com; https://www.novusbio.com; https://www.abcam.com; https://rockland-inc.com; https://www.thermofisher.com; or https://www.biolegend.com. Specificity of antibodies has also been validated by the authors in previous publications, including Doi et al., J Clin Endocrinol Metab, 2014, doi: 10.1210/jc.2013-3279, for HSD3B1; Yamamura et al., Mol Cell Endocrinol, 2014, doi: 10.1016/j.mce.2013.09.014, for Hsd3b6; Matsuo et al., Science, 2003, doi: 10.1126/science.1086271, for Per2; Tanaka et al., J Histochem Cytochem, 2011, doi: 10.1369/0022155411411090, for BrdU.

Eukaryotic cell lines

Policy information about cell lines

Cell line source(s)	H295R cells (ATCC, CRL-2128)
Authentication	The cell line was authenticated by the supplier, and also confirmed by growth condition and morphology by us.
Mycoplasma contamination	The cell line is negative for mycoplasma contamination.
Commonly misidentified lines (See ICLAC register)	No commonly misidentified cell line was used.

Animals and other organisms

Policy information about studies involving animals; ARRIVE guidelines recommended for reporting animal research

Laboratory animals	For analysis of wild type mice, C57BL/6J line was used. All transgenic and mutant mice (Hsd3b6 floxed mice; CAG-FLPe mice, CAG-Cre mice, K14-Cre mice, Bmal1-deficient mice, and PER2::LUC mice) were backcrossed to C57BL/6J. We used newborn male mice (P0) and adult female (2 months old) and male (2-25 months old) mice of specified genotype. Unless otherwise mentioned, all mice were maintained at 21°C ± 1°C with 30% ± 10% relative humidity, three animals per cage on a 12-hr-light/12-hr-dark cycle (lights on 8:00, lights off 20:00), food and water ad libitum. For evaporative dry eye test, mice were placed in a constant humidity of ~15% for 1 h.
Wild animals	This study did not involve wild animals.
Field-collected samples	This study did not involve samples collected from the field.
Ethics oversight	All mouse experiments were conducted in compliance with the Ethical Regulations of Kyoto University and performed under protocols approved by the Animal Care and Experimentation Committee of Kyoto University and Institutional Animal Care and Use Committee of RIKEN Kobe Branch.

Note that full information on the approval of the study protocol must also be provided in the manuscript.

Human research participants

Policy information about studies involving human research participants

Population characteristics	Meibography: Twelve normal, non-dry eye/non-MGD volunteers were involved in this study; three young women (mean age: 31.7 ± 2.3 SD years), 3 young men (32.6 ± 3.2 years), 3 elderly women (63.7 ± 3.1 years), and three elderly men (66.0 ± 4.0 years), and all subjects were native Japanese. Non-dry eye/non-MGD was diagnosed according to each of the Japanese diagnostic criteria (Tsubota et al., Ocul Surf, 2017, doi: 10.1016/j.jtos.2016.09.003). Human eyelid specimens: A formalin-fixed, paraffin-embedded human eyelid sample obtained from a post-mortem 53-year-old male donor who had no history of eye-related diseases was obtained from the Department of Frontier Medical Science and Technology for Ophthalmology, the Kyoto Prefectural University of Medicine, Japan. A formalin-fixed eyelid tissue sample from a post-mortem 34-year-old female donor who had no history of eye-related diseases was purchased from the Science Care, Inc. (Phoenix, AZ, USA).
Recruitment	Meibography: The following subjects were excluded from the study; tobacco smokers, contact lens wearers, and subjects with any eye and/or systemic disease, or who were taking medication at the time of the study. All subjects were joined in this study as a volunteer basis. There was no self-selection bias on these volunteer subjects who were native Japanese; i.e., of Asian ethnicity. Human eyelid specimens: the specimens were incidentally obtained, thus there was no selection bias.
Ethics oversight	The meibography was performed at the Kyoto City Hospital (KCH), which is affiliated with the Kyoto Prefectural University of Medicine (KPUM), Japan. This study was approved by the Institutional Review Boards of KCH and KPUM and performed in accordance with the principles of the Declaration of Helsinki. Written informed consent was obtained from all volunteers in this study. Immunohistochemical investigation of human eyelid specimens was performed at Kyoto University. This study protocol was approved by the Institutional Review Boards of both KPUM and Kyoto University.

Note that full information on the approval of the study protocol must also be provided in the manuscript.

Flow Cytometry

Plots

Confirm that:

- [x] The axis labels state the marker and fluorochrome used (e.g. CD4-FITC).
- [x] The axis scales are clearly visible. Include numbers along axes only for bottom left plot of group (a 'group' is an analysis of identical markers).
- [x] All plots are contour plots with outliers or pseudocolor plots.
- [x] A numerical value for number of cells or percentage (with statistics) is provided.

Methodology

Sample preparation	To obtain Itgav(+);CD45(−) meibomian gland cells from WT and Hsd3b6−/− mice, we used four mice per replicate, with two replicates for both genotypes. Tarsal plates were incubated in Hank's Balanced Salt Solution containing 0.25% Trypsin and 50 µg/mL DNaseI at 37 °C for 15 min. After minced, tissues were dissociated in phosphate-buffered saline (PBS) containing 20% fetal bovine serum (FBS) and 0.5 mM ethylenediaminetetraacetic acid (EDTA) and filtered through 70-µm strainer. Cell were resuspended in ice-cold PBS containing 2% FBS, 0.5 mM EDTA, and 50 µg/mL bovine serum albumin and blocked with anti-mouse CD16/32 antibody (BioLegend, 101302, 1:10 dilution) for 10 min. Cells were labelled with anti-CD45-APC/Cyanine7 (BioLegend, 104106, 1:100 dilution) and anti-Itgav-PE (BioLegend, 103116, 1:100 dilution) for 1 h and additionally incubated with DAPI for 5 min. Cell sorting was performed using a FACSAria II Flow cytometer with FACSDiva software (BD Biosciences).
Instrument	FACSAria II Flow cytometer (BD Biosciences)
Software	FACSDiva software (BD Biosciences)
Cell population abundance	For sorting for RNA-seq, at least 10,000 cells were collected.
Gating strategy	Cell suspensions were gated on FSC-A vs SSC-A to discard cell debris. Dead cells were excluded using DAPI staining. Itgav(+); CD45(−) cells were then gated followed by exclusion of doublets (FSC-H vs FSC-W and SSC-H vs SSC-W). Positive and negative gates were determined using unstained controls. Fluorophores were chosen to minimize spectral overlap.

☒ Tick this box to confirm that a figure exemplifying the gating strategy is provided in the Supplementary Information.
