# Supplementary figures and images for: Intracrine activity involving NAD-dependent circadian steroidogenic activity governs age-associated meibomian gland dysfunction
Source: Nat Aging. 2022 Feb 10;2(2):105–14. doi: 10.1038/s43587-021-00167-8 (PMC10154200; doi:10.1038/s43587-021-00167-8)

**Source Data Fig. 3**

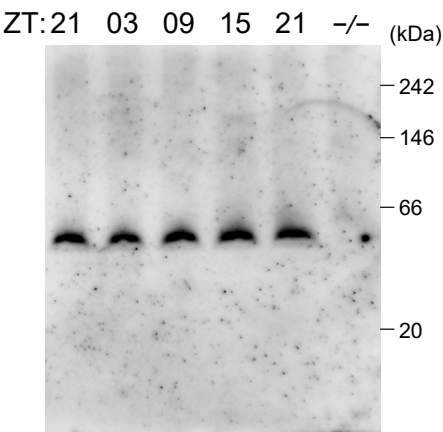

**Source Data Fig. 3** | Uncropped western blot image of Fig. 3g.

Supplement: Supplementary file 7 — Source Data Fig. 3 Unprocessed western blot data. [file 43587_2021_167_MOESM7_ESM.pdf]
